# Tumor‐Derived LAMB3 Drives Immunosuppressive LRRC15^+^ Fibroblast Formation During Pancreatic Ductal Adenocarcinoma Development

**DOI:** 10.1002/advs.202520029

**Published:** 2026-05-19

**Authors:** Xuqing Shi, Hangqi Liu, Jianru Sun, Xiaoding Liu, Xinping Jv, Longyun Chen, Yuhan Zhang, Hui Zhang, Xudong Xing, Ruiyu Li, Xinyi Ke, Jun Wang, Xianglin Yin, Bohan Liu, Qixian Liu, Yuan Wang, Junliang Lu, Shiyi Liu, Junyi Pang, Yumeng Cai, Menghua Dai, Fan Bai, Huanwen Wu, Zhiyong Liang

**Affiliations:** ^1^ Department of Pathology State Key Laboratory of Common Mechanism Research for Major Diseases Peking Union Medical College Hospital Chinese Academy of Medical Sciences & Peking Union Medical College Beijing China; ^2^ Beijing Institute of Genomics Chinese Academy of Sciences and China National Center for Bioinformation Beijing China; ^3^ Department of General Surgery Peking Union Medical College Hospital Chinese Academy of Medical Sciences & Peking Union Medical College Beijing China; ^4^ Biomedical Pioneering Innovation Center (BIOPIC) Peking‐Tsinghua Center for Life Sciences State Key Laboratory of Metabolic Dysregulation & Prevention and Treatment of Esophageal Cancer School of Life Sciences Peking University (PKU) Beijing China; ^5^ Peking University Beijing‐Tianjin‐Hebei Biomedical Pioneering Innovation Center Tianjin China

**Keywords:** cancer‐associated fibroblasts, cellular crosstalk, laminin, leucine‐rich repeat‐containing 15, pancreatic ductal adenocarcinomas

## Abstract

Pancreatic ductal adenocarcinoma (PDAC) is characterized by a highly immunosuppressive and desmoplastic tumor microenvironment (TME) that limits the efficacy of immunotherapy. However, the evolution of this immunosuppressive TME and the underlying mechanisms remain incompletely understood. Here, we construct a dynamic single‐cell atlas spanning uninvolved adjacent pancreatic tissue (UNIN), intraductal papillary mucinous neoplasm (IPMN), and PDAC. We confirm the stepwise establishment of an immunosuppressive milieu, accompanied by the emergence of LRRC15^+^ fibroblasts as determinants. Functional assays further identify tumor‐derived LAMB3 as a regulator of LRRC15^+^ fibroblast differentiation. Mechanistically, LAMB3 promotes FOSL2‐dependent transcriptional activation of LRRC15 through the ITGB1/FAK/MAPK signaling axis, ultimately suppressing T cell cytotoxicity. Orthotopic models reveal that LAMB3 overexpression increases the LRRC15 positive area and impairs T cell cytotoxicity, whereas FAK inhibition partially reverses these effects. In parallel, LAMB3 knockdown reduces the LRRC15 positive area and improves the efficacy of PD‐1 blockade. Moreover, glycolytic reprogramming in PDAC ductal cells upregulates LAMB3 expression and correlates with increased LRRC15^+^ fibroblast enrichment. Clinically, co‐enrichment of LAMB3^+^ PDAC ductal cells and LRRC15^+^ fibroblasts is associated with inferior overall survival. Collectively, our findings define a dynamic ductal‐fibroblast‐immune multicellular axis underlying PDAC pathogenesis and provide insights into potential therapeutic strategies.

## Graphical abstract

A single‐cell atlas of pancreatic ductal adenocarcinoma development reveals progressive ductal‐fibroblast‐immune crosstalk. Tumor‐derived LAMB3 drives the formation of immunosuppressive LRRC15^+^ fibroblasts through the ITGB1/FAK/MAPK/FOSL2 signaling. Glycolytic reprogramming upregulates LAMB3 and correlates with LRRC15^+^ fibroblast enrichment. Clinical cohorts link the LAMB3‐LRRC15 axis to poor survival, while LAMB3 knockdown enhances immunotherapy efficacy in vivo, highlighting a potential therapeutic vulnerability.

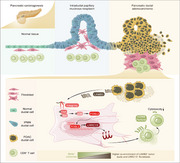

## 1 Introduction

Pancreatic ductal adenocarcinoma (PDAC) is one of the most aggressive malignancies, with a five‐year overall survival (OS) rate of approximately 10% [1]. Although immunotherapy has markedly improved outcomes in multiple solid tumors, patients with PDAC derive only modest clinical benefit [2, 3]. Increasing evidence suggests that this therapeutic resistance is not solely attributable to tumor cell‐intrinsic mechanisms, but is also closely linked to the unique tumor microenvironment (TME) of PDAC, which is characterized by significant immunosuppression and extensive desmoplasia [4, 5]. Therefore, elucidating the evolution of the immunosuppressive TME and the underlying mechanisms during PDAC development may provide novel therapeutic vulnerabilities [6, 7].

PDAC development is a multistage process from the normal pancreas to precursor lesions and ultimately to invasive carcinoma [8]. Intraductal papillary mucinous neoplasm (IPMN) is a bona fide macroscopic precursor lesion of PDAC, providing an opportunity to dissect microenvironmental remodeling during pancreatic tumorigenesis. Analyses of multistage pancreatic lesions have revealed a stepwise establishment of the immunosuppressive milieu during disease progression, accompanied by progressive T cell dysfunction and tumor‐promoting macrophage polarization [9, 10, 11, 12]. Importantly, this immune evolution is paralleled by stromal remodeling.

Among stromal components, fibroblasts are key regulators of both immunosuppression and desmoplasia [13, 14]. In PDAC, fibroblasts have been broadly classified into myofibroblastic, inflammatory, and antigen‐presenting subsets based on distinct transcriptional and functional features [15, 16]. Advances in single‐cell RNA sequencing (scRNA‐seq) have further expanded this framework, revealing additional fibroblast phenotypes and highlighting their remarkable plasticity [17]. Among fibroblast subpopulations, LRRC15^+^ fibroblasts have emerged as a functionally important subset within the myofibroblastic lineage [18, 19]. LRRC15 (leucine‐rich repeat‐containing 15) is a transmembrane protein with restricted expression in most normal adult tissues, but is enriched in pathological stromal contexts [20]. Previous studies have associated LRRC15^+^ fibroblasts with poor responses to immunotherapy across multiple tumor types, including bladder cancer and glioblastoma [19, 21]. Studies in mouse models of PDAC have demonstrated that LRRC15^+^ fibroblasts impair T cell function, while depleting them enhances the efficacy of immunotherapy [18]. Together, these findings suggest that LRRC15^+^ fibroblasts are increasingly regarded not merely as stromal markers, but as a functionally relevant fibroblast phenotype associated with immune evasion. However, their temporal emergence during human PDAC development, their relationship to immune dysfunction, and the upstream mechanisms governing their formation remain incompletely understood.

Mechanistically, fibroblast phenotypic plasticity is shaped by microenvironmental cues [22]. Specifically, TGFβ signaling has been reported to promote the formation of LRRC15^+^ fibroblasts in PDAC and glioblastoma, highlighting the role of extrinsic signaling in fibroblast remodeling [18]. Nevertheless, TGFβ alone is unlikely to fully account for the upstream regulatory network modulating LRRC15^+^ fibroblast differentiation. Given the close interactions between tumor ductal cells and fibroblasts, tumor‐derived paracrine factors, including extracellular matrix (ECM)‐associated signaling molecules, may represent plausible drivers of this process [22, 23, 24, 25].

Laminins are key components of the basement membrane and ECM, providing structural support while also functioning as signaling molecules. LAMB3 encodes the laminin β3 chain, a subunit of laminin heterotrimers. Previous studies have implicated LAMB3 in PDAC cell proliferation, invasion, and migration through the PI3K‐AKT and EGFR cascades [26, 27, 28]. However, whether LAMB3 participates in fibroblast remodeling, particularly in driving LRRC15^+^ fibroblast formation and thereby contributing to impaired anti‐tumor immunity, remains unclear in PDAC. In addition, metabolic rewiring is a hallmark of PDAC [29]. The role of metabolic rewiring in regulating LAMB3 expression and fibroblast plasticity also remains largely unknown.

To address these questions, we integrated scRNA‐seq, spatial transcriptomics, and clinicopathological validation to comprehensively delineate the coordinated evolution of ductal and microenvironmental components during PDAC development with a particular focus on the dynamics and immunoregulatory roles of LRRC15^+^ fibroblasts. We employed in vitro models to investigate the role of tumor ductal cell‐derived LAMB3 in LRRC15^+^ fibroblast differentiation and the underlying molecular mechanisms. Orthotopic mouse models were further used to assess the in vivo functional relevance of the LAMB3‐LRRC15 axis and its impact on responsiveness to PD‐1 blockade. We also investigated the upstream contribution of metabolic rewiring to LAMB3 expression and the clinical relevance of the LAMB3‐LRRC15 axis in PDAC. Collectively, our study reveals dynamic ductal‐fibroblast‐immune crosstalk underpinning PDAC pathogenesis and identifies a potential therapeutic axis.

## 2 Results

### 2.1 scRNA‐seq Reveals a Stepwise Immunosuppressive Microenvironment During Human PDAC Development

**FIGURE 1:**
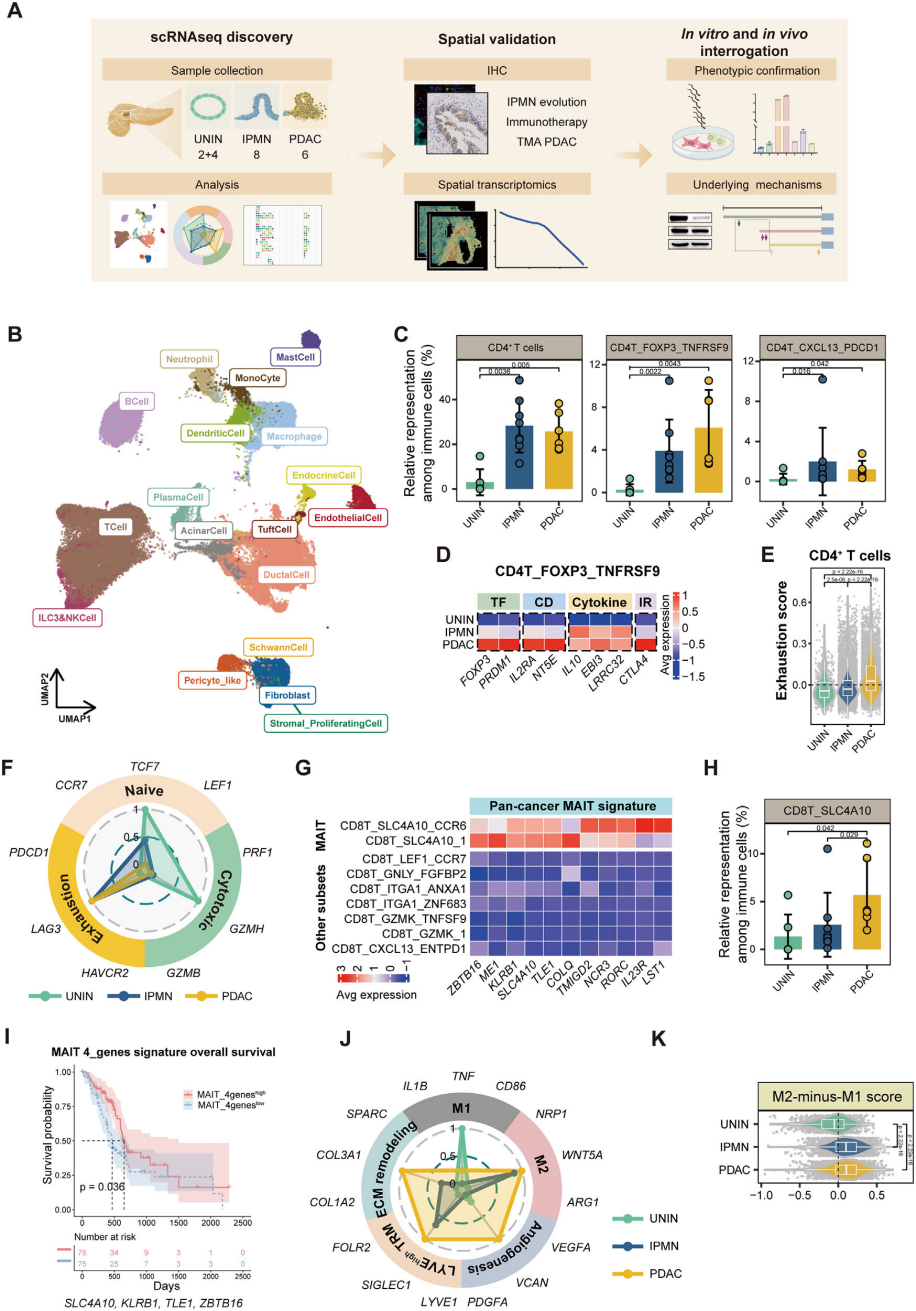
Immune landscape dynamics across the PDAC development continuum. (A) Schematic overview of the study design. (B) UMAP visualization of 82 760 high quality cells annotated by major cell types. (C) Bar plots showing the relative representation of CD4^+^ T cells and their subpopulations among total immune cells in UNIN (n = 6), IPMN (n = 8), and PDAC (n = 6) tissues. (D) Heatmap comparing relative expression levels of selected genes in the CD4T_FOXP3_TNFRSF9 across UNIN, IPMN, and PDAC stages. (E) Violin plot showing the exhaustion scores of CD4^+^ T cells at different stages (UNIN: n = 701; IPMN: n = 9845; PDAC: n = 5284). (F) Radar plot illustrating selected signature scores in CD8^+^ T cells across pathological stages. Axis represents the normalized score of each signature scaled to 0–1. (G) Heatmap visualizing the relative expression of genes from pan‐cancer MAIT signature in CD8^+^ T cell subpopulations. (H) Bar plot showing the proportion of CD8T_SLC4A10 subset among total immune cells in UNIN, IPMN, and PDAC tissues (UNIN: n = 6; IPMN: n = 8; PDAC: n = 6). (I) Kaplan–Meier survival curve comparing MAIT_4genes^high^ (n = 75) and MAIT4_genes^low^ (n = 75) groups in TCGA‐PAAD cohort. (J) Radar plot showing selected signatures scored in macrophages across pathological stages. Axis represents the normalized score of each signature scaled to 0–1. (K) Violin plot visualizing M2 score minus M1 score in macrophages across different stages (UNIN: n = 1055; IPMN: n = 2454; PDAC: n = 2268). Statistical analysis was performed using the two‐tailed Wilcoxon rank‐sum test (C, E, H, and K), log‐rank test (I). For C and H, data are shown as mean ± SD.

To delineate the cellular dynamics of PDAC development, we performed scRNA‐seq on treatment‐naive pancreatic specimens, including 6 uninvolved pancreatic tissues (4 samples from public datasets) [30, 31], 8 intraductal papillary mucinous neoplasms, and 6 PDAC samples (Figure 1A). Following stringent quality control and doublet removal, a total of 82 760 high‐confidence cells were retained for further analysis (Figure S1A–C). We distinguished epithelium, stroma, and immunity according to their canonical markers (Figure 1B; Figure S1D–F). To explore the immune dynamics, T cells and myeloid cells were further characterized.

CD4^+^ T cells and their subsets, CD4T_FOXP3_TNFRSF9 and CD4_CXCL13_PDCD1, were already relatively enriched at the IPMN stage (Figure 1C; Figure S2A,B). The CD4T_FOXP3_TNFRSF9 subset, characterized by high expression of *FOXP3* and *TNFRSF9*, was annotated as regulatory T cells (Tregs) [32]. Compared with CD4T_FOXP3_TNFRSF9 in the UNIN tissue, their counterparts in IPMN and PDAC lesions exhibited a more activated Treg effector phenotype, evidenced by increased expression of *FOXP3*, *PRDM1* (alias *BLIMP1*), *IL2RA*, *LRRC32*, and *CTLA4* (Figure 1D). Along disease progression from UNIN to IPMN, and ultimately to PDAC, CD4^+^ T cells presented progressively dysfunctional features marked by increased expression of immune checkpoint molecules, such as *HAVCR2* and *TIGIT*, together with a gradual decline in the naive signature score (Figure 1E; Figure S2D,E).

Next, CD8^+^ T cells were categorized into 9 distinct subpopulations (Figure S2A). Among them, CD8_LEF1_CCR7 exhibited the highest naive signature, CD8T_GNLY_FGFBP2 displayed the strongest cytotoxic activity, and CD8T_CXCL13_ENTPD1 was annotated as an exhausted T cell subset based on its highest exhaustion signature (Figure S2C). During PDAC carcinogenesis, CD8^+^ T cells displayed reduced expression of naive markers (*TCF7*, *SELL*, *LEF1*) and effector molecules (*PRF1*, *GNLY*, *NKG7*, *GZMB*), while exhibiting increased expression of the inhibitory receptor (*LAG3*) and exhaustion‐associated transcription factors (TFs) (*TOX*, *SOX4*, *BATF*) (Figure 1F; Figure S2F) [33]. These findings indicate a progressive impairment of T cell function during PDAC development. Notably, the SLC4A10^high^ subsets, comprising CD8T_SLC4A10_CCR6 and CD8T_SLC4A10_1, shared transcriptional profiles with pan‐cancer mucosal‐associated invariant T (MAIT) cells [34], and were therefore annotated as MAIT cells (Figure 1G). In the scRNA‐seq dataset, MAIT cells showed increased relative representation in both IPMN and PDAC lesions compared with uninvolved tissues (Figure 1H). To assess the clinical relevance of MAIT cells, we reanalyzed the TCGA‐PAAD cohort. A four‐gene MAIT signature derived from our dataset was associated with improved OS (Figure 1I), consistent with the prognostic value of the previously reported pan‐cancer MAIT signature (Figure S2G).

Among macrophages, Mac_FOLR2 was characterized by distinctively high expression of *LYVE1*, *SIGLEC1*, and *FOLR2*, closely mirroring tumor‐permissive LYVE1^high^ tissue‐resident macrophages (TRM) (Figure S2A,H) [35, 36]. In alignment with the pro‐fibrotic response of LYVE1^high^ TRM described in a recent study [35], Mac_FOLR2 demonstrated potent ECM remodeling activity compared to Mac_SPP1 (Figure S2J). Furthermore, both LYVE1^high^ TRM and ECM remodeling scores were increased in macrophages as IPMN evolved (Figure 1J; Figure S2I). Meanwhile, macrophages exhibited a shift from M1 to M2 polarization, evidenced by diminished expression of M1‐associated genes (*IL1B*, *TNF*, *NOS2*), alongside escalating trends in both the M2 score and the M2‐minus‐M1 score, and enhanced expression of M2‐associated genes (*STAT6*, *IL10*) (Figure 1K; Figure S2K). Moreover, the angiogenic capacity of macrophages was strikingly elicited in the PDAC group. Taken together, macrophages underwent profound transcriptional reprogramming during PDAC carcinogenesis to facilitate the immune evasion of tumor cells.

### 2.2 Fibroblasts Acquire an Immunosuppressive LRRC15^+^ Fibroblast Phenotype During Human PDAC Development

**FIGURE 2:**
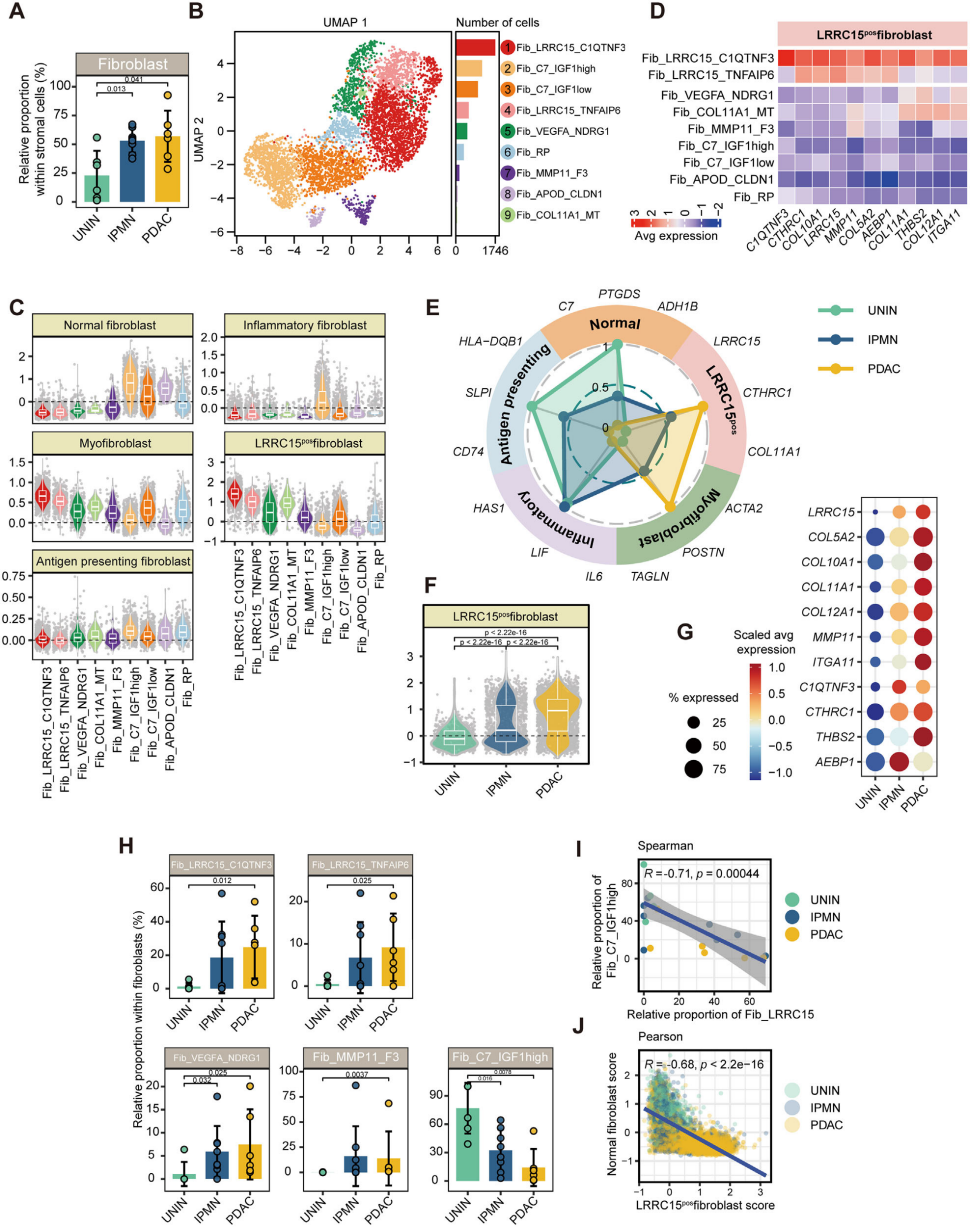
Functional and compositional dynamics of fibroblast subpopulations across different stages. (A) Bar plot revealing relative proportion of fibroblasts within the stromal compartment across UNIN, IPMN, and PDAC in the scRNA‐seq dataset (UNIN: n = 6; IPMN: n = 8; PDAC: n = 6). (B) UMAP plot depicting the distribution of fibroblast subpopulations, with cell counts shown on the right. (C) Violin plots revealing phenotypic scores of fibroblast subpopulations. (D) Heatmap showing the expression patterns of LRRC15^pos^fibroblast signature genes across fibroblast subpopulations. (E) Radar plot showing selected signatures scored in fibroblasts across different pathological stages. Axis represents the normalized score of each signature scaled to 0–1. (F) Violin plot showing LRRC15^pos^fibroblast signature scores in fibroblasts across different stages (UNIN: n = 611; IPMN: n = 1899; PDAC: n = 3086). (G) Dot plot displaying scaled average expression of LRRC15^pos^fibroblast signature genes in fibroblasts across groups. The dot size represents the proportion of cells expressing the genes in each group, and color intensity reflects expression levels. (H) Bar plots showing the relative proportions of fibroblast subsets within the fibroblast compartment across UNIN, IPMN, and PDAC samples in the scRNA‐seq dataset (UNIN: n = 6; IPMN: n = 8; PDAC: n = 6). (I) Scatter plot showing the Spearman correlation between the relative proportions of Fib_LRRC15 and Fib_C7_IGF1high across samples in the scRNA‐seq dataset. Shading represents 95% confidence intervals. (J) Scatter plot depicting the Pearson correlation between LRRC15^pos^fibroblast score and the normal fibroblast score in each fibroblast. Statistical analysis was performed using a two‐tailed Wilcoxon rank‐sum test (A, F, and H). For A and H, data are shown as mean ± SD.

Observing the increased relative representation of fibroblasts in the scRNA‐seq data from IPMN and PDAC samples, we next investigated fibroblast state dynamics across disease progression (Figure 2A,B). Fibroblasts were classified into 9 transcriptionally distinct subsets (Figure S3A,B). To better position these subsets within the established PDAC fibroblast taxonomy, we compared their transcriptional patterns. The Fib_C7_IGF1high subset, distinguished by complement factors (*C7* and *CFD*) secretion, exhibited a pronounced normal pancreatic fibroblast signature together with an inflammatory fibroblast phenotype (Figure 2C) [19]. In contrast, Fib_LRRC15_C1QTNF3, Fib_LRRC15_TNFAIP6, Fib_VEGFA_NDRG1, Fib_COL11A1_MT, Fib_MMP11_F3, and Fib_C7_IGF1low, were enriched for myofibroblast features, indicating that these subsets largely represent myofibroblasts. Fib_APOD_CLDN1 exhibited a significant antigen‐presenting fibroblast signature, supporting its classification as an antigen‐presenting fibroblast subset. Fib_RP was characterized by high *RPS12* expression, consistent with active ribosome biogenesis, and exhibits elevated scores for both antigen‐presenting fibroblast and myofibroblast signatures, suggesting a ribosome biogenesis‐active fibroblast state that may span both myofibroblast and antigen‐presenting populations. Notably, Fib_LRRC15, encompassing Fib_LRRC15_C1QTNF3 and Fib_LRRC15_TNFAIP6 subsets, closely resembled immunosuppressive LRRC15^+^ myofibroblasts reported previously [19], as reflected by the highest LRRC15^pos^fibroblast signature score together with significant myofibroblast signature enrichment (Figure 2D; Figure S3C).

During IPMN initiation and progression, fibroblasts exhibited diminished normal, inflammatory, and antigen‐presenting properties (Figure 2E–G; Figure S3E). Concurrently, they acquired augmented myofibroblast and LRRC15^pos^fibroblast phenotypes, along with marked upregulation of antigen (*LRRC15*), collagens (*COL12A1*, *COL10A1*, *COL5A2*), matrix metallopeptidase (*MMP11*), and integrin (*ITGA11*) [37]. Similarly, reanalysis of the CPTAC‐PDAC cohort revealed increased LRRC15 expression and a higher LRRC15^pos^fibroblast signature in PDAC tissue than in normal tissue (Figure S3F) [38]. These data highlight the functional reprogramming of fibroblasts during PDAC carcinogenesis.

In line with the expression and signature dynamics observed above, fibroblast subclusters with prominent LRRC15^pos^fibroblast and myofibroblast features, including Fib_LRRC15_C1QTNF3, Fib_LRRC15_TNFAIP6, showed progressively increased relative representation within the fibroblast compartment during progression to PDAC. Other subsets with myofibroblast features, including Fib_VEGFA_NDRG1 and Fib_MMP11_F3, also exhibited higher relative proportions in PDAC, whereas the relative proportion of the normal fibroblast‐like Fib_C7_IGF1high subcluster showed a marked decrease (Figure 2H).

**FIGURE 3:**
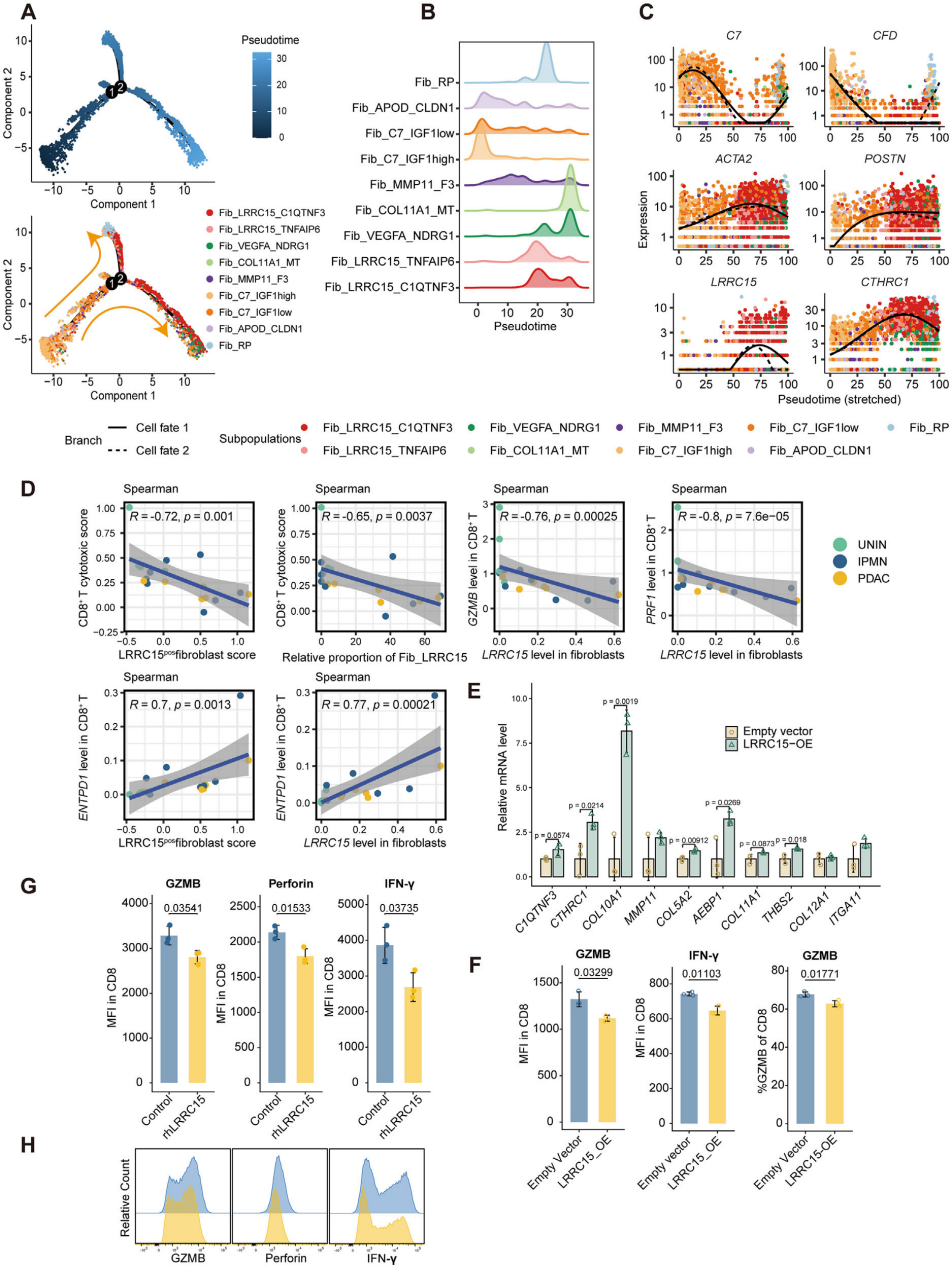
LRRC15^+^ fibroblasts in PDAC tissue attenuate T cell cytotoxicity. (A) Pseudo‐time trajectory plots of fibroblasts, colored by pseudo‐time (top) and subpopulation identity (bottom) in a two‐dimensional space. Each point denotes a single cell. Arrow indicates potential fibroblast differentiation pathways. (B) Ridgeline plots illustrating the distribution of different subpopulations along the pseudo‐time axis. (C) Two‐dimensional plots depicting the dynamic expression of marker genes for normal fibroblasts, myofibroblasts, and LRRC15^pos^fibroblasts along pseudo‐time. (D) Spearman correlation analyses were performed across samples in the scRNA‐seq dataset to assess associations between fibroblast‐ and CD8^+^ T cell‐related features, including functional signature scores, relative cell proportions, and marker gene expression levels. Shading represents 95% confidence intervals. (E) RT‐qPCR analysis of relative mRNA expression of LRRC15^pos^fibroblast signature genes in LRRC15‐OE and empty vector control primary CAFs (n = 3 per group). (F) Flow cytometric analysis of cytotoxic activity in activated primary human CD8^+^ T cells co‐cultured with control or LRRC15‐OE CAFs. MFI of GZMB and IFN‐γ, as well as the percentage of GZMB^+^ CD8^+^ T cells, are shown (n = 3 in each group). (G) Flow cytometric analysis of activated primary human CD8^+^ T cells treated with rhLRRC15. MFI of GZMB, perforin, and IFN‐γ in CD8^+^ T cells are shown (n = 3 in each group). (H) Representative flow cytometry histograms showing expression levels of GZMB, perforin, and IFN‐γ in CD8^+^ T cells under control and rhLRRC15 treatment conditions in G. Statistical analysis is performed using two‐tailed Student's *t* test (E, F, and G). For E, F, and G, data are shown as mean ± SD.

To elucidate the relationships among fibroblast subpopulations, we performed correlation analyses of their relative representation, functional properties, and representative markers. The relative proportion of Fib_C7_IGF1high was inversely correlated with that of Fib_LRRC15 (Figure 2I; Figure S3G). Likewise, the normal fibroblast and LRRC15^pos^fibroblast phenotypes were significantly negatively correlated, paralleled by an inverse association between their representative markers, *C7* and *LRRC15* (Figure 2J; Figure S3H). These associations were consistent with the possibility of a transition toward LRRC15^+^ fibroblast states. To further examine this possibility, we employed Monocle 2 to reconstruct a pseudo‐time trajectory of fibroblast state progression [39]. Fib_C7_IGF1high was positioned at the early stage of the pseudo‐time trajectory, whereas Fib_LRRC15_C1QTNF3, Fib_LRRC15_TNFAIP6, Fib_VEGFA_NDRG1 and Fib_COL11A1_MT were predominantly distributed at later stages (Figure 3A,B). Consistently, pseudo‐time progression was accompanied by downregulation of *C7* and concomitant upregulation of *LRRC15* (Figure 3C).

We next investigated whether the relative representation of Fib_LRRC15 during human PDAC development was associated with impaired CD8^+^ T cell function. CD8^+^ T cell cytotoxic activity was inversely associated with both the LRRC15^pos^fibroblast score and the relative proportion of Fib_LRRC15, consistent with significant negative correlations between *LRRC15* expression in fibroblasts and *GZMB* and *PRF1* expression in CD8^+^ T cells (Figure 3D). Furthermore, both the LRRC15^pos^fibroblast score and *LRRC15* expression in fibroblasts were positively correlated with the *ENTPD1* (encoding CD39) expression, a marker associated with T cell dysfunction in CD8^+^ T cells [40, 41]. The association between the impaired cytotoxic activity of CD8^+^ T cells and the emergence of LRRC15^+^ fibroblasts led us to hypothesize that LRRC15^+^ fibroblasts may contribute to the suppression of T cell cytotoxic function in human PDAC.

To test this hypothesis in vitro, we used primary cancer‐associated fibroblasts (CAFs) derived from human PDAC samples as an experimentally tractable fibroblast model. Rather than assuming that these cells were equivalent to bona fide LRRC15^+^ fibroblasts in vivo, we used them as a cellular background to generate LRRC15‐overexpressing (LRRC15‐OE) fibroblasts. LRRC15‐OE CAFs showed increased expression of genes comprising the LRRC15^pos^fibroblast signature, supporting the establishment of an LRRC15^+^ fibroblast‐like phenotype in LRRC15‐OE CAFs compared with vector control CAFs (Figure 3E). We then co‐cultured activated primary human CD8^+^ T cells with LRRC15‐OE CAFs, and observed the impaired CD8^+^ T cell cytotoxic function, as evidenced by reduced GZMB and IFN‐γ expression and a decreased proportion of GZMB^+^CD8^+^ T cells (Figure 3F). Similarly, treatment with recombinant human LRRC15 (rhLRRC15) contributed to the downregulation of GZMB, perforin (encoded by *PRF1*), and IFN‐γ in activated CD8^+^ T cells (Figure 3G,H). Together, these results underscore that LRRC15^+^ fibroblasts attenuate the cytotoxic capacity of CD8^+^ T cells in human PDAC in vitro.

### 2.3 LAMB3‐Mediated Cellular Interactions are Linked to LRRC15^+^ Fibroblast Formation

**FIGURE 4:**
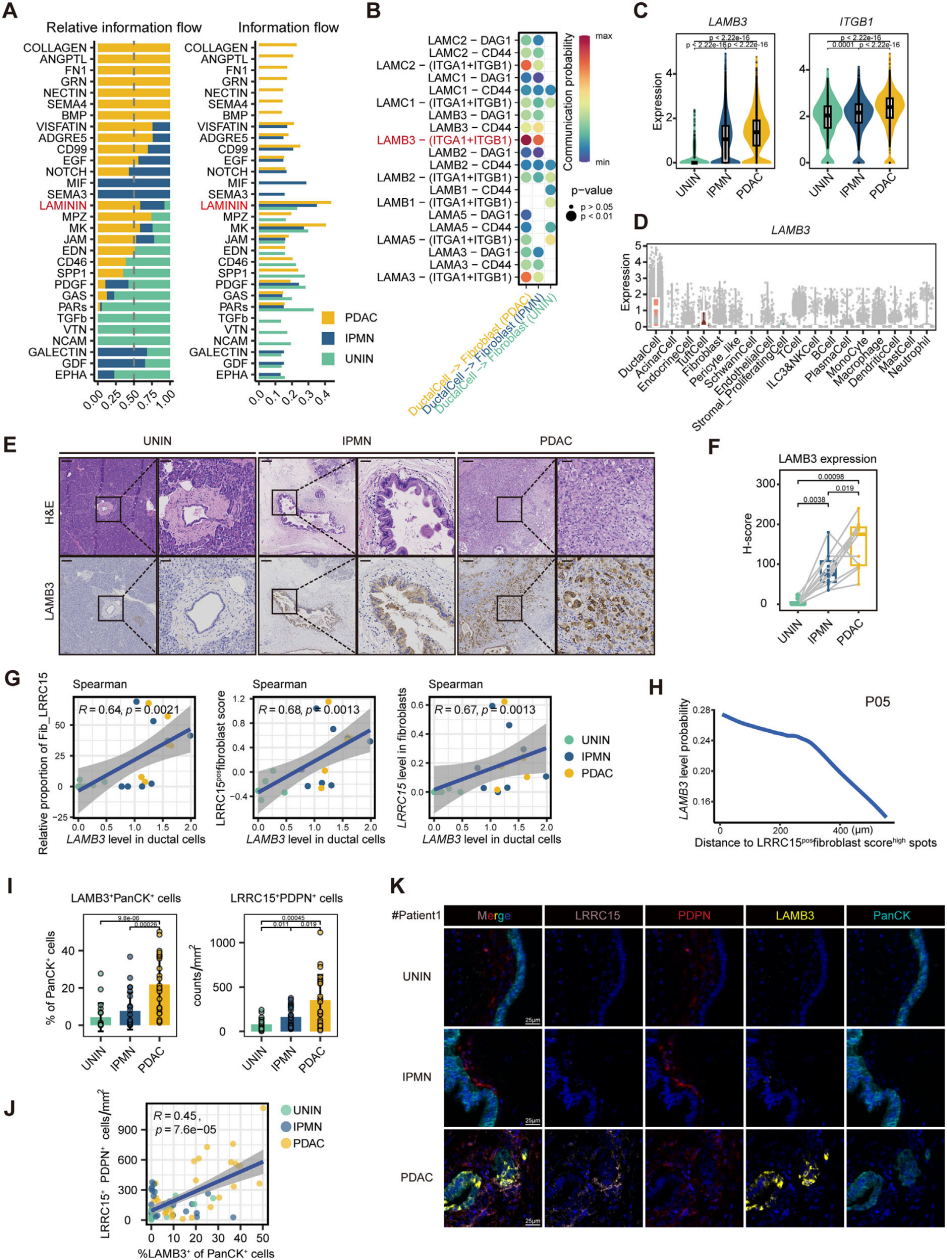
LAMB3^+^ PDAC ductal cells and LRRC15^+^ fibroblasts are in close proximity within PDAC tissue. (A) Bar plots comparing predicted information flow from ductal cells to fibroblasts across disease stages. (B) Dot plot revealing the intensity of LAMININ pathway‐mediated signaling from ductal cells to fibroblasts across disease stages. Dot color and size indicate communication probability and statistical significance, respectively. (C) Violin plots illustrating the expression levels of *LAMB3* (UNIN: n = 522; IPMN: n = 9100; PDAC: n = 7671) in ductal cells and *ITGB1* (UNIN: n = 611; IPMN: n = 1899; PDAC: n = 3086) in fibroblasts by stages. (D) Box plot showing *LAMB3* expression across major cell types. (E) Representative IHC images showing LAMB3 protein across stages in the same patient. Scale bars: low magnification, 200 µm; high magnification, 50 µm. (F) Paired box plot illustrating LAMB3 H‐score in ductal cells across distinct disease stages (UNIN: n = 11; IPMN: n = 11; PDAC: n = 11). (G) Scatter plots showing the Spearman correlations between *LAMB3* expression in ductal cells and LRRC15^+^ fibroblast features across samples in the scRNA‐seq dataset. Shading represents the 95% confidence interval. (H) Line diagram showing the spatial distance between LRRC15^pos^fibroblast signature and LAMB3 expression in P05 sample. The x‐axis represents distance from spots with high LRRC15^pos^fibroblast signature, and the y‐axis indicates *LAMB3* expression probability. (I) Bar plots quantifying the fraction of LAMB3^+^ cells among PanCK^+^ epithelial cells and the density of LRRC15^+^PDPN^+^ fibroblasts per mm^2^ across stages (UNIN: n = 20; IPMN: n = 25; PDAC: n = 25). (J) Spearman correlation between LAMB3^+^/PanCK^+^ epithelial cell fraction and LRRC15^+^PDPN^+^ fibroblasts per mm^2^ in each ROI. Shading represents 95% confidence intervals. (K) Representative mIHC images showing spatial proximity of LRRC15^+^ fibroblasts to LAMB3^+^ epithelial cells within PDAC tissue. Scale bar, 25 µm. Statistical analysis was performed using two‐tailed Wilcoxon rank‐sum test (C and I) and paired two‐tailed Wilcoxon signed‐rank test (F). For C, F, and I, data are shown as mean ± SD.

To investigate microenvironmental factors contributing to the development of the immunosuppressive LRRC15^+^ fibroblasts within the pancreas during carcinogenesis, we utilized the CellChat toolkit to examine the kinetics of fibroblast interactions with other major cell types [42]. This analysis revealed that fibroblasts were engaged in a broad spectrum of intercellular communication (Figure S4A). Among all predicted signaling pathways targeting fibroblasts, the FN1, COLLAGEN, and LAMININ pathways were progressively activated in a stepwise fashion from uninvolved tissue to IPMN stage and ultimately to PDAC stage. Notably, LAMININ signaling originated exclusively from epithelial and stromal compartments, with no contribution from immune cells (Figure S4B). Interestingly, within the LAMININ pathway targeting fibroblasts, LAMB3‐mediated interactions were confined to the epithelium. Subsequently, we dissected the crosstalk flow from ductal cells to fibroblasts and found that LAMININ signaling was significantly activated during IPMN initiation and its progression to PDAC. Among these interactions, LAMB3‐mediated crosstalk exhibited a particularly marked intensification (Figure 4A,B).

Consistently, the expression of *LAMB3* in ductal cells and its receptors *ITGB1* in fibroblasts increased progressively in a stage‐dependent manner (Figure 4C; Figure S4C), with a positive correlation between the two genes (Figure S4D). Moreover, reanalysis of two bulk RNA‐seq datasets from the studies by Hiraoka et al. and Liffers et al. corroborated an upregulation of *LAMB3* expression during IPMN progression [43, 44], in line with elevated LAMB3 protein levels in the tumor samples of the CPTAC‐PDAC cohort (Figure S4E) [38].

Not surprisingly, in accordance with the cellular origins of LAMB3‐mediated interactions, *LAMB3* expression was primarily localized to ductal cells (Figure 4D; Figure S4F). Immunohistochemistry (IHC) analysis further demonstrated its predominant expression in ductal cells rather than the stromal compartments, with significantly elevated expression in PDAC ductal cells compared to those in uninvolved and IPMN tissues (Figure 4E,F). These findings underscore the considerable potential of LAMB3 as a tumor‐specific biomarker in PDAC.

Subsequently, we investigated the role of ductal cell‐derived LAMB3‐mediated interactions in shaping fibroblast phenotypic transitions. Across uninvolved, pre‐cancerous, and cancerous tissues, *LAMB3* expression in ductal cells was positively correlated with the features of LRRC15^+^ fibroblasts while displaying an inverse association with normal pancreatic fibroblast features (Figure 4G; Figure S4G). External validation using the CPTAC‐PDAC cohort yielded similar results (Figure S5A). Moreover, spatial transcriptomic profiling of tissues from patients P04 and P05 demonstrated that *LAMB3* expression declined with increasing distance from regions enriched in LRRC15^pos^fibroblast signature (Figure 4H; Figure S5B–F). To further assess the spatial co‐localization, multiplex IHC (mIHC) analysis was employed in 70 pancreatic regions from 5 IPMN‐associated PDAC patients. The densities of both LAMB3^+^ epithelial cells and LRRC15^+^ fibroblasts progressively increased during PDAC development. This was accompanied by a pronounced regional correlation between the densities of two cell types, highlighting their co‐enrichment during PDAC carcinogenesis (Figure 4I,J). Importantly, LAMB3^+^ epithelial cells were frequently located in close proximity to LRRC15^+^ fibroblasts in PDAC tissue (Figure 4K; Figure S5G). Collectively, these data demonstrate that ductal cell‐derived LAMB3 is strongly associated with LRRC15^+^ fibroblasts across transcriptional, proteomic, and spatial dimensions.

### 2.4 Co‐Existence of LAMB3^+^ PDAC Ductal Cells and LRRC15^+^ Fibroblasts Indicates Unfavorable Clinical Outcomes

**FIGURE 5:**
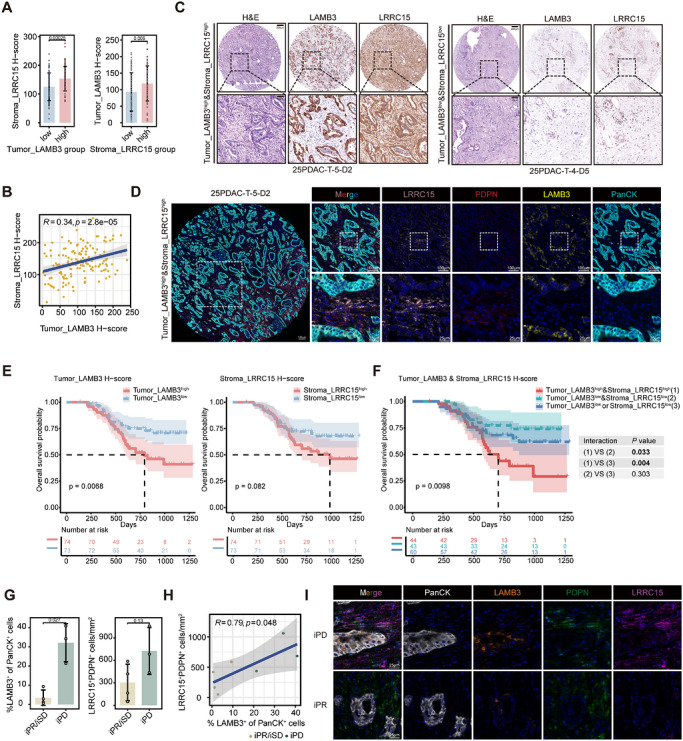
Distribution and clinical relevance of co‐occurrence of LAMB3^+^ ductal cells and LRRC15^+^ fibroblasts in PDAC. (A) Bar plots showing stroma_LRRC15 H‐scores in patients stratified by tumor_LAMB3 H‐score (left) (low: n = 73; high: n = 74) and tumor_LAMB3 H‐scores in patients stratified by stroma_LRRC15 H‐score (right; low: n = 73; high: n = 74) in a PDAC TMA cohort comprising 147 patients with resected PDAC. (B) Pearson correlation between tumor_LAMB3 and stroma_LRRC15 H‐scores across the TMA PDAC cohort. Shading represents 95% confidence intervals. (C) Representative IHC images from two patients with either concordantly high or low tumor_LAMB3 and stroma_LRRC15 H‐scores. Scale bars: low magnification, 200 µm; high magnification, 50 µm. (D) Representative mIHC images from the patient with concordantly high tumor_LAMB3 and stroma_LRRC15 H‐scores shown in C. Scale bars: low magnification, 100 µm; high magnification, 25 µm. (E) Kaplan–Meier survival curves stratified by tumor_LAMB3 H‐score (left) or stroma_LRRC15 H‐score (right) in TMA PDAC cohort. (F) Kaplan–Meier curve showing overall survival based on combined tumor_LAMB3 and stroma_LRRC15 H‐scores in TMA‐PDAC cohort. (G) Bar plots quantifying the fraction of LAMB3^+^ cells among PanCK^+^ epithelial cells and the density of LRRC15^+^PDPN^+^ fibroblasts per mm^2^ across response groups (iPR/iSD: n = 4; iPD: n = 3). (H) Spearman correlation between LAMB3^+^/PanCK^+^ epithelial cell fraction and LRRC15^+^PDPN^+^ fibroblasts per mm^2^ in each case. Shading represents 95% confidence intervals. (I) Representative mIHC images depicting the spatial co‐localization of LRRC15^+^ fibroblasts and LAMB3^+^ epithelial cells in the immunotherapy cohort. Scale bars, 25 µm. Statistical analysis was performed using two‐tailed Wilcoxon rank‐sum test (A), two‐tailed Student's *t* test (G), log‐rank test (E and F), and Tarone–Ware test (F). For A and G, data are shown as mean ± SD.

The progressive spatial accumulation of both LAMB3^+^ ductal cells and LRRC15^+^ fibroblasts during PDAC pathogenesis prompted us to investigate whether this co‐existence stratifies clinical outcomes. In a tissue microarray (TMA) cohort comprising 147 patients with resected PDAC, LAMB3 expression in tumor ductal epithelium and LRRC15 expression in the stroma were quantified using the H‐score method. IHC staining of serial sections revealed that patients with higher tumor_LAMB3 H‐score exhibited elevated stroma_LRRC15 levels. Likewise, patients with higher stroma_LRRC15 H‐score exhibited elevated tumor_LAMB3 levels (Figure 5A). In addition, tumor_LAMB3 and stromal_LRRC15 H‐scores were positively correlated (Figure 5B). This co‐expression pattern observed in IHC was also validated by mIHC (Figure 5C,D; Figure S6A). Moreover, Kaplan–Meier analysis showed that patients with high tumor_LAMB3 H‐score had significantly worse OS (Figure 5E). Meanwhile, patients with high stroma_LRRC15 H‐score tended to have shorter OS. Notably, patients with concurrent high expression of tumor_LAMB3 and stroma_LRRC15 had worse OS than patients with either dual‐low expression or low expression of only one marker (Figure 5F). In line with these results, multivariate Cox regression analysis supported the hypothesis that tumor_LAMB3 overexpression alone and its combined overexpression with stroma_LRRC15 independently predicted adverse OS in PDAC (Figure S6B–D). Consistently, CPTAC‐PDAC cohort validated the prognostic significance of the co‐expression of LAMB3 and LRRC15^pos^fibroblast signature (Figure S6E,F).

To explore the potential therapeutic relevance of the spatial co‐enrichment of LAMB3^+^ epithelial cells and LRRC15^+^ fibroblasts, we analyzed a pilot immunotherapy cohort comprising 7 PDAC patients. After postoperative recurrence and/or metastatic progression during follow‐up, patients were enrolled in a clinical trial and received combined anti‐CD73 and anti‐PD‐1 immunotherapy. Archival surgical specimens collected prior to immunotherapy were used for spatial analysis. Cases with immune progressive disease (iPD) showed a trend toward higher densities of LAMB3^+^ epithelial cells (Figure 5G). Moreover, mIHC analysis revealed spatial proximity between LAMB3^+^ epithelial cells and LRRC15^+^ fibroblasts in iPD cases (Figure 5H,I). Although limited by the small sample size, these exploratory findings suggest a possible association between the LAMB3‐LRRC15 axis and response to the combined anti‐CD73 and anti‐PD‐1 immunotherapy.

### 2.5 LAMB3 Drives LRRC15^+^ Fibroblast Formation via the ITGB1/FAK/MAPK/FOSL2 Signaling

**FIGURE 6:**
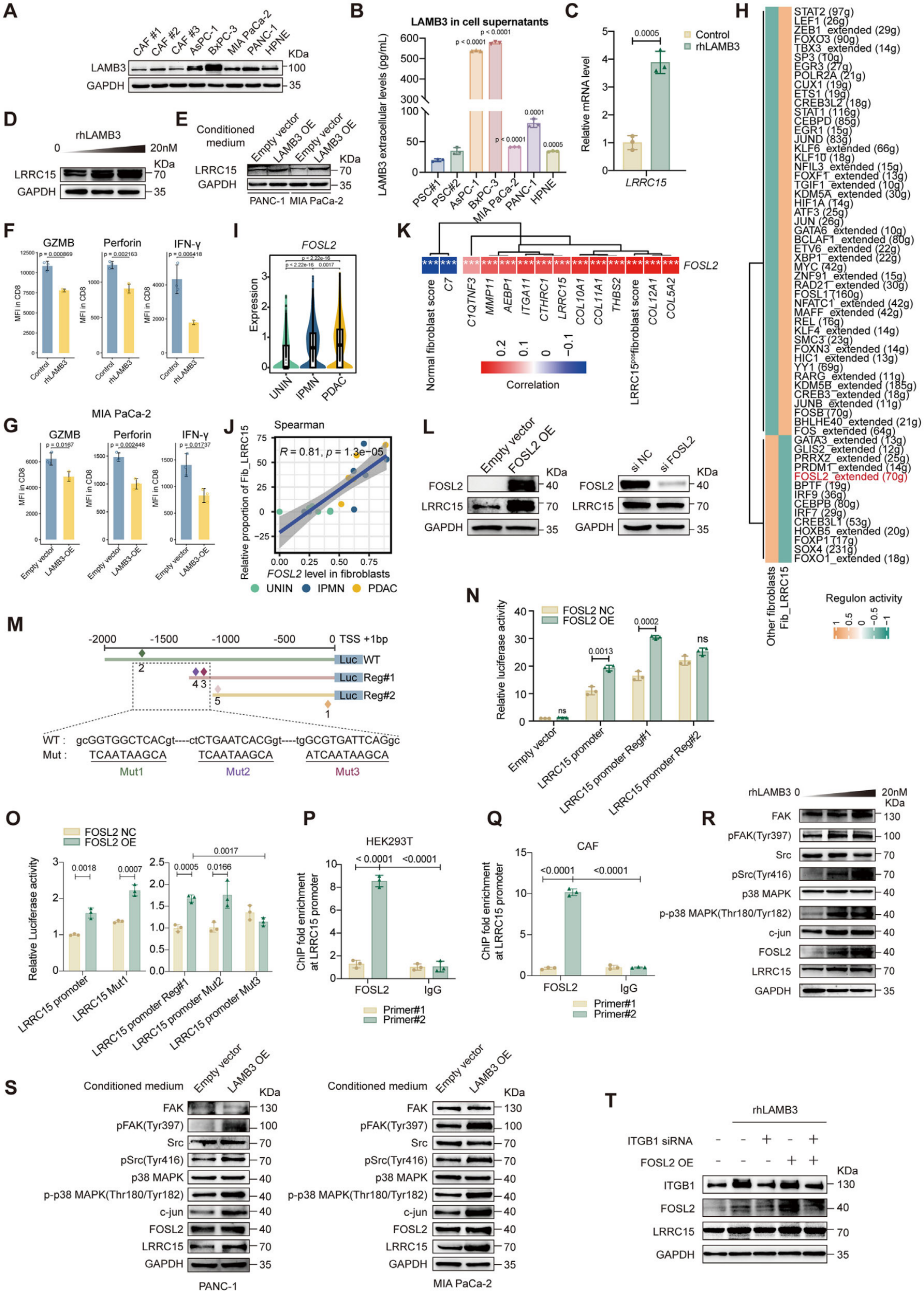
LAMB3 derived from PDAC ductal cells promotes LRRC15 expression in fibroblasts by activating the ITGB1/FAK/MAPK/FOSL2 pathway. (A) Western blot analysis of LAMB3 protein level in various cell lines. (B) ELISA‐based quantification of secreted LAMB3 level from different cells (n = 3 in each group). (C) RT‐qPCR analysis of *LRRC15* mRNA levels in primary CAFs treated with rhLAMB3 (n = 3 per group). (D) Western blot analysis of LRRC15 protein level in CAFs treated with varying concentrations of rhLAMB3. (E) Western blot analysis of LRRC15 level in CAFs treated with conditioned medium from LAMB3‐OE PANC‐1 and MIA PaCa‐2. (F) Flow cytometric analysis of cytotoxic activity in activated primary human CD8^+^ T cells directly co‐cultured with CAFs pretreated with rhLAMB3 (n = 3 per group). (G) human CD8^+^ T cells were directly co‐cultured with CAFs pretreated with conditioned medium from LAMB3‐OE MIA‐PaCa‐2, and the cytotoxicity was assessed by flow cytometry (n = 3 in each group). (H) Heatmap comparing TF regulon activity between Fib_LRRC15 and other fibroblast subpopulations. (I) Violin plot illustrating *FOSL2* expression in fibroblasts across stages (UNIN: n = 611; IPMN: n = 1899; PDAC: n = 3086). (J) Scatter plot showing the Spearman correlation between *FOSL2* expression in fibroblasts and the relative proportion of Fib_LRRC15 among total fibroblasts across samples in the scRNA‐seq dataset. Shading represents the 95% confidence interval. (K) Heatmap displaying Pearson correlations between *FOSL2* expression and fibroblast phenotypes across stages. (L) Western blot analysis of FOSL2 and LRRC15 protein levels in CAFs following FOSL2 overexpression or siRNA‐mediated FOSL2 knockdown. (M) Schematic diagram depicting truncated luciferase reporter constructs for the LRRC15 promoter and engineered mutant FOSL2‐binding sites within the LRRC15 promoter. (N) Luciferase activity in HEK293T cells transfected with distinct LRRC15 promoter fragments under FOSL2 overexpression or control conditions (n = 3 in each group). (O) Luciferase activity in HEK293T cells transfected with constructs harboring wild‐type or mutant FOSL2‐binding sites within LRRC15 promoter fragments under FOSL2 overexpression vs. control conditions (n = 3 in each group). (P, Q) Bar plots showing FOSL2 enrichment at LRRC15 promoter in HEK293T cells (P) and CAFs (Q). ChIP assays were performed using anti‐IgG and anti‐FOSL2. Subsequent qPCR analysis was conducted using primers targeting distinct regulatory regions within the LRRC15 promoter, and GAPDH was used as negative control. Primer #1 was specifically designed for putative FOSL2 binding site 2, while primer #2 was designed for putative binding sites 3 and 4 (n = 3 in each group). (R) Western blot analysis of ITGB1/FAK/MAPK/FOSL2 signaling activation in CAFs treated with varying concentrations of rhLAMB3. (S) Western blot analysis of ITGB1/FAK/MAPK/FOSL2 pathway activation in CAFs treated with conditioned medium from PANC‐1 or MIA PaCa‐2 overexpressing LAMB3. (T) Western blot of LRRC15 expression in CAFs with siITGB1 or FOSL2‐OE following rhLAMB3 treatment. Statistical analysis was performed using two‐tailed Student's *t* test (B, C, F, G, N, O, P, and Q) and two‐tailed Wilcoxon rank‐sum test (I). For B, C, F, G, N, O, P, and Q, data are shown as mean ± SD. ^*^ *p* < 0.05, ^**^ *p* < 0.01, ^***^ *p* < 0.001.

To disentangle the molecular mechanism underlying the observed co‐localization and potential interaction between LAMB3^+^ PDAC ductal cells and LRRC15^+^ fibroblasts, we first examined the expression and secretion of LAMB3. LAMB3 expression was markedly elevated in PDAC cell lines, such as AsPC‐1 and BxPC‐3, compared with normal human pancreatic epithelial nestin‐expressing (HPNE) cells and CAFs, consistent with our IHC observations (Figure 6A). Moreover, ELISA assays further showed that PDAC cell lines secreted substantially higher levels of soluble LAMB3 than CAFs (Figure 6B).

Based on these findings, we hypothesized that LAMB3 derived from PDAC ductal cells promotes the formation of LRRC15^+^ fibroblasts. To test this hypothesis, CAFs were treated with recombinant human LAMB3 (rhLAMB3), which led to increased *LRRC15* mRNA expression (Figure 6C). In parallel, rhLAMB3 treatment also upregulated multiple genes within the LRRC15^pos^fibroblast signature, including *C1QTNF3*, *COL5A2*, and *MMP11*, indicating the induction of an LRRC15^pos^fibroblast phenotype in CAFs (Figure S7A). Moreover, rhLAMB3 induced a dose‐dependent increase in LRRC15 protein expression in CAFs (Figure 6D). Consistently, conditioned medium from LAMB3‐OE PDAC cell lines also increased LRRC15 protein level in CAFs (Figure 6E; Figure S7B). Together, these findings support a role for LAMB3 in promoting LRRC15^+^ fibroblast induction in vitro.

Given the suppressive effect of LRRC15^+^ fibroblasts on T cell cytotoxic function, we next investigated whether LAMB3 could indirectly modulate T cell‐mediated anti‐tumor immunity through fibroblast reprogramming. To this end, CAFs were first pretreated with rhLAMB3 and then co‐cultured with activated primary human CD8^+^ T cells. Flow cytometric analysis showed that rhLAMB3‐pretreated fibroblasts significantly reduced GZMB, perforin, and IFN‐γ expression, as well as the corresponding positive subset in CD8^+^ T cells (Figure 6F; Figure S7C). Consistently, CAFs pretreated with conditioned medium from LAMB3‐OE MIA PaCa‐2 or PANC‐1 cells also impaired CD8^+^ T cell cytotoxic function after co‐culture, as evidenced by reduced GZMB, perforin, and IFN‐γ expression, together with decreased proportions of cytotoxic subsets (Figure 6G; Figure S7D,E). These results support the hypothesis that ductal cell‐derived LAMB3 drives LRRC15^+^ fibroblast formation, thereby suppressing T cell cytotoxic function in PDAC.

Subsequently, we explored the mechanism underpinning LAMB3‐induced LRRC15 expression in fibroblasts. Single‐cell regulatory network inference and clustering (SCENIC) analyses unveiled that the TF regulons *GLIS2*, *SOX4*, *CREB3L1*, and *FOSL2* were significantly activated in LRRC15^+^ fibroblasts compared to their negative counterparts (Figure 6H) [45, 46]. FOSL2 is a component of the activator protein 1 (AP‐1) TF complex. Meanwhile, elevated *FOSL2* expression in fibroblasts during carcinogenesis was strongly associated with a shift toward LRRC15^+^ fibroblast states, as supported by its correlations with fibroblast subpopulation relative proportions, phenotype scores, and marker gene expression (Figure 6I–K; Figure S7F). Similar results were observed in the external datasets (Figure S7G,H). These findings prompted the question of whether FOSL2 regulates the development of LRRC15^+^ fibroblasts. Genetic perturbation experiments supported this relationship: FOSL2 overexpression led to an increase in LRRC15 expression, whereas FOSL2 knockdown substantially reduced LRRC15 expression (Figure 6L; Figure S7I), suggesting FOSL2 acts as an upstream regulator of LRRC15 in fibroblasts.

We next asked whether FOSL2 directly regulates LRRC15 transcription. To test this, we generated a series of LRRC15 promoter deletion constructs based on predicted FOSL2‐binding motifs from the JASPAR database (Figure 6M) [47]. Luciferase reporter assays revealed robust transcriptional activity in FOSL2‐OE HEK293T cells with either the full‐length LRRC15 promoter or the Region 1 fragment (Figure 6N), strongly implicating binding sites 3 and 4 in FOSL2‐mediated LRRC15 regulation. Notably, mutation of these putative FOSL2‐binding sites markedly abolished promoter activity (Figure 6O). Consistently, ChIP‐qPCR in HEK293T cells showed FOSL2 enrichment of at the LRRC15 promoter (Figure 6P). Importantly, to provide cell‐type‐specific validation, we performed ChIP‐qPCR in CAFs and confirmed similar enrichment of FOSL2 at the LRRC15 promoter, particularly at binding sites 3 and 4 (Figure 6Q). Together, these results highlight that FOSL2 directly binds to the LRRC15 promoter and transcriptionally regulates LRRC15 in fibroblasts.

We then sought to define the upstream signaling pathway by which LAMB3 activates FOSL2. Integrins, as putative receptors for LAMB3, have been demonstrated to mediate mechanosensory activation of FAK [48]. Meanwhile, the MAPK signaling pathway serves as a pivotal downstream effector of FAK signaling [49]. Additionally, the AP‐1 TF complex has been recognized as a crucial target within the MAPK cascade [50]. Therefore, it is theoretically plausible that LAMB3 elicits LRRC15 expression in fibroblasts through the ITGB1/FAK/MAPK/FOSL2 signaling pathway. The genes relevant to this pathway were progressively upregulated in fibroblasts during the evolution of IPMN and positively correlated with both LAMB3^+^ ductal cells and LRRC15^+^ fibroblasts in internal and external datasets (Figure S7J–N). Functionally, both rhLAMB3 and conditioned medium from LAMB3‐OE PDAC cells enhanced expression of FOSL2 and LRRC15, concomitant with the phosphorylation and activation of FAK/MAPK/AP‐1 signaling cascade in CAFs (Figure 6R,S). Notably, ITGB1 knockdown abrogated the rhLAMB3‐induced LRRC15 expression, which could be rescued by FOSL2 overexpression (Figure 6T; Figure S7O). These results support a mechanistic model in which LAMB3 activates ITGB1/FAK/MAPK signaling, culminating in FOSL2‐dependent LRRC15 transcription and immunosuppressive fibroblast differentiation.

### 2.6 LAMB3 Drives Stromal‐Immune Remodeling and Limits Response to PD‐1 Blockade in Orthotopic PDAC

**FIGURE 7:**
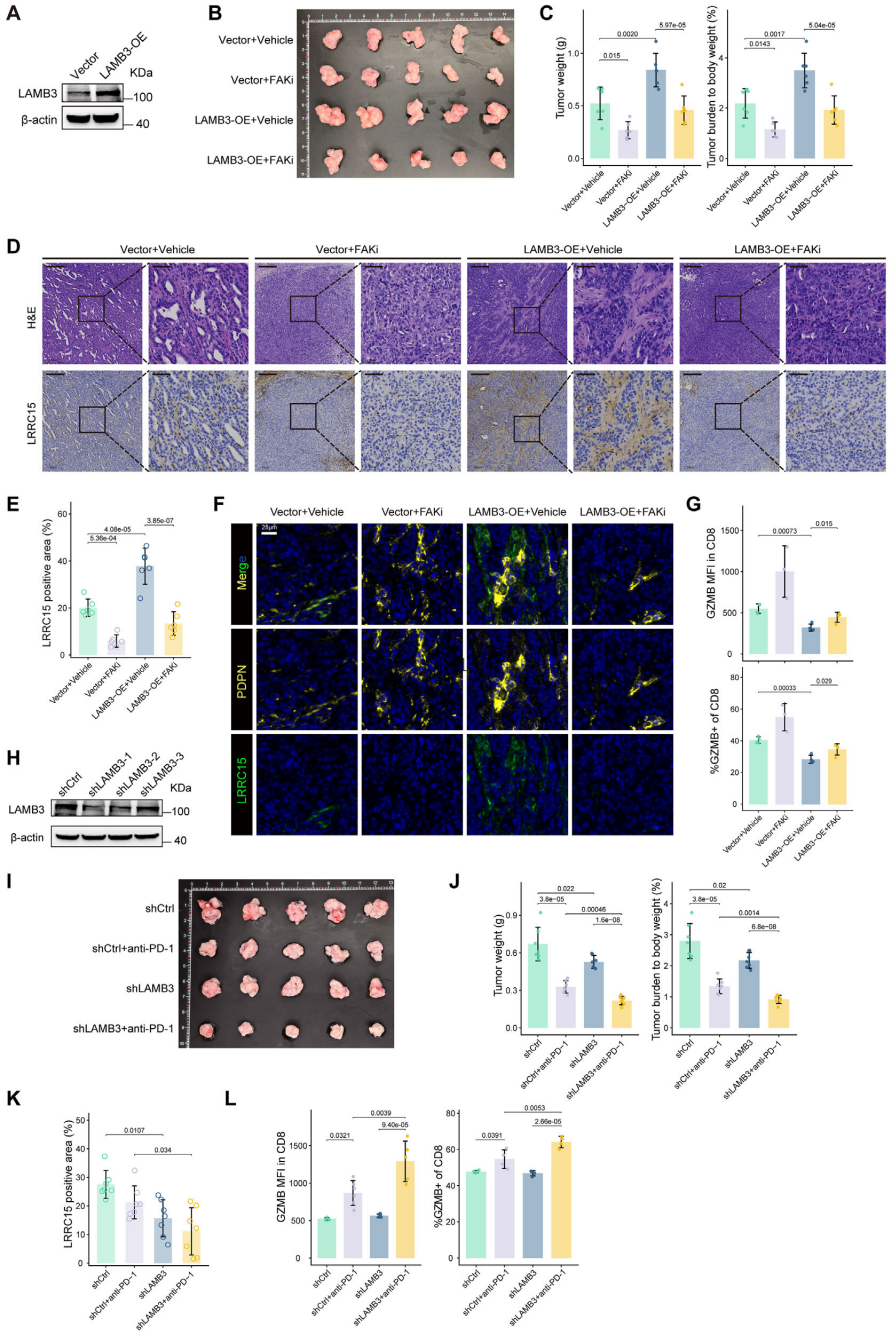
LAMB3 drives stromal‐immune remodeling and impairs the cytotoxic activity of CD8^+^ T cells in vivo. (A) Western blot analysis of LAMB3 protein expression in KPC cells with LAMB3‐OE. (B) Representative images of orthotopic tumors from mice implanted with vector control or LAMB3‐OE KPC cells and treated with vehicle or FAKi. (C) Tumor weight and tumor burden relative to body weight in the vector + vehicle (n = 6), vector + FAKi (n = 6), LAMB3‐OE + vehicle (n = 6), and LAMB3‐OE + FAKi (n = 6) groups. (D) Representative images of H&E staining and LRRC15 IHC of tumor sections from the vector + vehicle, vector + FAKi, LAMB3‐OE + vehicle, and LAMB3‐OE + FAKi groups. Scale bars: low magnification, 100 µm; high magnification, 25 µm. (E) Quantification of the LRRC15 positive area in tumor sections from the vector + vehicle (n = 6), vector + FAKi (n = 6), LAMB3‐OE + vehicle (n = 6), and LAMB3‐OE + FAKi (n = 6) groups. (F) Representative mIHC images showing PDPN and LRRC15 expression in tumor sections from the vector + vehicle, vector + FAKi, LAMB3‐OE + vehicle, and LAMB3‐OE + FAKi groups. Scale bar, 25 µm. (G) Flow cytometric analysis of GZMB MFI and the proportion of GZMB^+^ cells among tumor‐infiltrating CD8^+^ T cells in the vector + vehicle (n = 4), vector + FAKi (n = 3), LAMB3‐OE + vehicle (n = 4), and LAMB3‐OE + FAKi (n = 4) groups. (H) Western blot analysis confirming LAMB3 knockdown in KPC cells transduced with shRNAs targeting LAMB3. shLAMB3‐2 was used for subsequent in vivo experiments. (I) Representative images of orthotopic tumors from mice implanted with shCtrl or shLAMB3 KPC cells and treated with or without anti‐PD‐1 antibody. (J) Tumor weight and tumor burden relative to body weight in the shCtrl (n = 7), shCtrl + anti‐PD‐1 (n = 7), shLAMB3 (n = 7), and shLAMB3 + anti‐PD‐1 (n = 7) groups. (K) Quantification of the LRRC15 positive area in the shCtrl (n = 7), shCtrl + anti‐PD‐1 (n = 7), shLAMB3 (n = 7), and shLAMB3 + anti‐PD‐1 (n = 7) groups. (L) Flow cytometric analysis of GZMB MFI and the proportion of GZMB^+^ cells among tumor‐infiltrating CD8^+^ T cells in the shCtrl (n = 4), shCtrl + anti‐PD‐1 (n = 4), shLAMB3 (n = 4), and shLAMB3 + anti‐PD‐1 (n = 4) groups. Statistical analysis was performed using one‐way ANOVA with Tukey's post hoc test (C, E, G, J, K, and L). For C, E, G, J, K, and L, data are shown as mean ± SD. The orthotopic mouse experiment was performed once.

To determine the role of LAMB3 in stromal‐immune remodeling in vivo, we established an orthotopic PDAC model in immunocompetent C57BL/6 mice using syngeneic KPC cells stably overexpressing LAMB3 (LAMB3‐OE) or carrying the corresponding vector control (Vector). To further interrogate the functional involvement of FAK signaling, tumor‐bearing mice were treated with the FAK inhibitor (FAKi) VS‐4718 or vehicle (Figure 7A).

Orthotopic tumors derived from the LAMB3‐OE group displayed a significantly greater tumor burden than those derived from the vector group, indicating that LAMB3 overexpression facilitates PDAC progression. Administration of VS‐4718 reduced tumor burden in both vector and LAMB3‐OE groups (Figure 7B,C). Similar findings were observed when tumor burden was normalized to body weight, further supporting the contribution of FAK signaling to LAMB3 overexpression‐driven tumor progression.

To assess stromal remodeling in vivo, we quantified the percentage of LRRC15 positive area in orthotopic tumors by IHC. LRRC15 staining was predominantly localized to the stromal compartment (Figure 7D). Compared with vector control tumors, LAMB3‐OE tumors showed a significant increase in the percentage of LRRC15 positive area (Figure 7E). Notably, FAK inhibition significantly attenuated the LAMB3 overexpression‐induced expansion of the LRRC15 positive area. mIHC showed that LRRC15 signal was predominantly localized to PDPN^+^ stromal cells, supporting that the LRRC15 positive area identified by IHC largely represented fibroblast‐rich stromal regions (Figure 7F).

To determine whether LAMB3 also affects tumor immunity, we performed flow cytometric analysis of tumor‐infiltrating T cells. Compared with vector control tumors, LAMB3‐OE tumors showed reduced GZMB median fluorescence intensity (MFI) in CD8^+^ T cells and a lower proportion of GZMB^+^ CD8^+^ T cells (Figure 7G; Figure S8). Importantly, in LAMB3‐OE tumors, FAK inhibition partially reversed the reduction in CD8^+^ T cell cytotoxic activity, as evidenced by increased GZMB MFI and a higher frequency of GZMB^+^ cells among CD8^+^ T cells than in vehicle‐treated LAMB3‐OE tumors. Collectively, these findings support a model in which LAMB3 promotes orthotopic PDAC progression, enhances stromal LRRC15 expression, and dampens CD8^+^ T cell cytotoxic activity, at least in part through FAK‐dependent mechanisms.

To investigate whether LAMB3 influences responsiveness to immunotherapy in vivo, we established orthotopic PDAC models using KPC cells with stable LAMB3 knockdown (shLAMB3) or the corresponding control (shCtrl) (Figure 7H). Anti‐PD‐1 treatment was initiated on day 10 after implantation. PD‐1 blockade reduced tumor burden in both shCtrl and shLAMB3 tumors, indicating that anti‐PD‐1 exerted measurable antitumor effects in this orthotopic model (Figure 7I,J). Notably, LAMB3 knockdown further enhanced the therapeutic efficacy of PD‐1 blockade, as evidenced by a pronounced reduction in both tumor weight and tumor‐to‐body weight ratio in PD‐1 blockade‐treated shLAMB3 tumors than in anti‐PD‐1‐treated shCtrl tumors.

In parallel, LAMB3 knockdown markedly reduced the percentage of LRRC15 positive area relative to shCtrl tumors both in the presence and absence of anti‐PD‐1 treatment, indicating that LAMB3 contributes to an increased stromal LRRC15‐positive area in vivo (Figure 7K).

Further flow cytometric analysis showed that PD‐1 blockade increased GZMB expression and the frequency of GZMB^+^ cells among CD8^+^ T cells in both shCtrl and shLAMB3 tumors (Figure 7L). Compared with PD‐1 blockade‐treated shCtrl tumors, PD‐1 blockade‐treated shLAMB3 tumors showed greater increases in GZMB expression, along with higher proportions of GZMB^+^ cells among CD8^+^ T cells. Taken together, these findings indicate that LAMB3 knockdown enhances immunotherapeutic responsiveness, accompanied by a reduced percentage of LRRC15‐positive area and improved CD8^+^ T cell cytotoxic activity.

### 2.7 Glycolytic Reprogramming of Tumoral Ductal Cells Promotes LAMB3 Upregulation During PDAC Development

**FIGURE 8:**
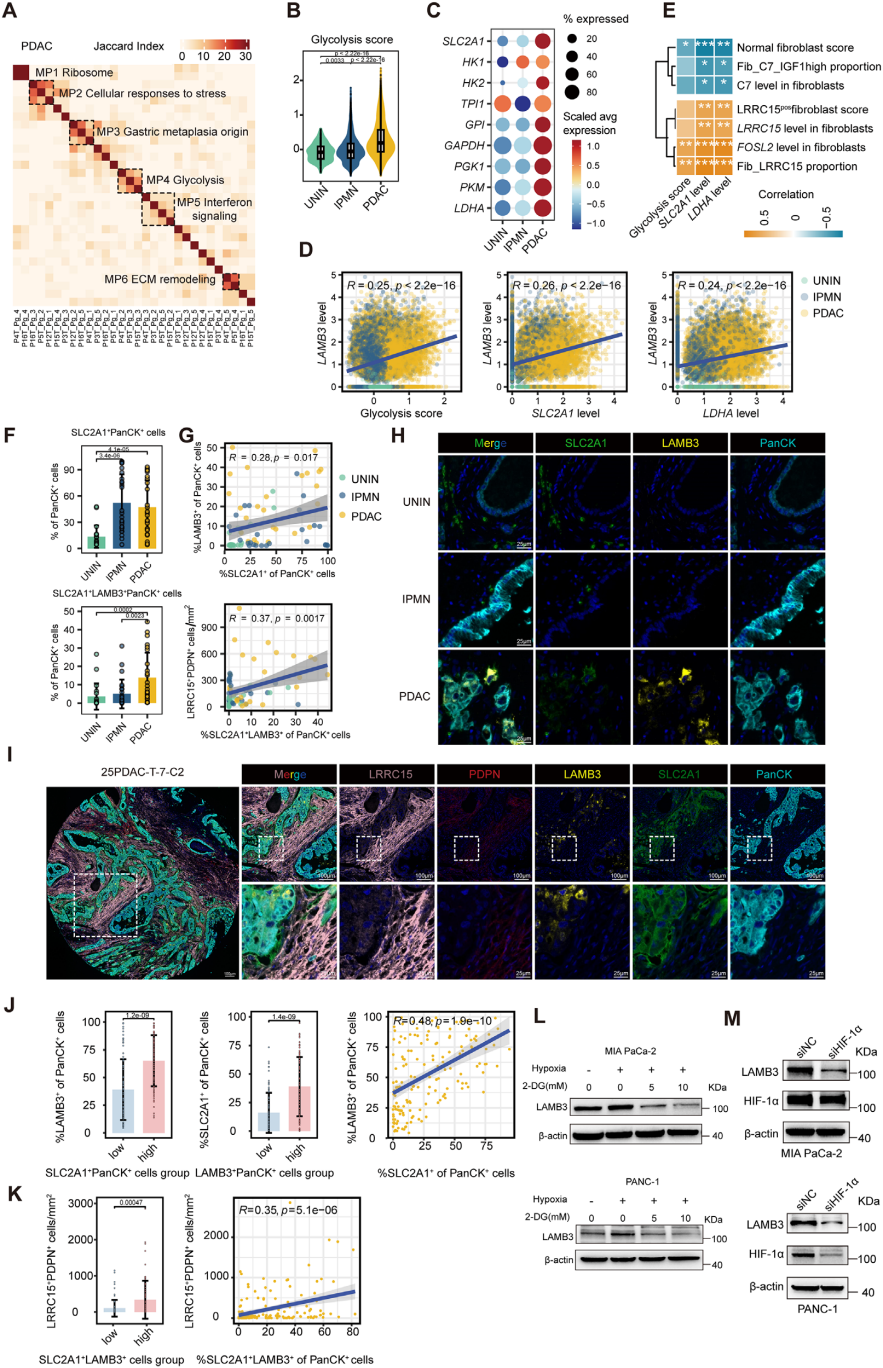
Glycolytic reprogramming in PDAC ductal cells upregulates LAMB3 expression in PDAC. (A) Heatmap of hierarchical clustering based on Jaccard similarity between transcriptional programs identified by non‐negative matrix factorization in PDAC tissue samples. (B) Violin plot depicting glycolysis scores of ductal cells across different stages (UNIN: n = 522; IPMN: n = 9100; PDAC: n = 7671). (C) Dot plot showing scaled average expression of glycolysis‐associated genes in ductal cells. Dot size represents the proportion of expressing cells in each group. Color represents expression intensity. (D) Pearson correlation between LAMB3 expression and glycolytic activity in individual ductal cells. (E) Heatmap displaying Spearman correlations between glycolytic features in ductal cells and fibroblast phenotypes. (F) Bar plots depicting fractions of SLC2A1^+^ cells and SLC2A1^+^LAMB3^+^ cells among PanCK^+^ epithelial cells across stages (UNIN: n = 20; IPMN: n = 25; PDAC: n = 25). (G) Spearman correlation between LAMB3^+^ and SLC2A1^+^ cell fractions out of PanCK^+^ cells (top), and between LRRC15^+^ fibroblast density per mm^2^ and SLC2A1^+^LAMB3^+^/PanCK^+^ cells per ROI (bottom). Shading represents 95% confidence intervals. (H) Representative mIHC images showing the presence of SLC2A1^+^LAMB3^+^PanCK^+^ cells during PDAC development. (I) Representative mIHC images showing the co‐enrichment of SLC2A1^+^LAMB3^+^ epithelial cells and LRRC15^+^ fibroblasts in a patient from the TMA PDAC cohort. (J) Bar plots showing the fractions of LAMB3^+^ cells out of PanCK^+^ cells in patients stratified by the fractions of SLC2A1^+^ cells out of PanCK^+^ cells (left) (low: n = 79; high: n = 79) and the fractions of SLC2A1^+^ cells out of PanCK^+^ cells in patients stratified by the fractions of LAMB3^+^ cells out of PanCK^+^ cells (middle) (low: n = 79; high: n = 79) in TMA PDAC cohort. Scatter plot (right) depicting Pearson correlation between SLC2A1^+^ epithelial cells and LAMB3^+^ epithelial cells across patients in the TMA PDAC cohort. Shading represents 95% confidence intervals. (K) Bar plot (left) showing the density of LRRC15^+^ fibroblasts per mm^2^ in patients stratified by the fractions of SLC2A1^+^LAMB3^+^ cells out of PanCK^+^ cells (low: n = 79; high: n = 79). Scatter plot (right) depicting Pearson correlation between LRRC15^+^ fibroblast density per mm^2^ and the fractions of SLC2A1^+^LAMB3^+^ cells out of PanCK^+^ cells in TMA PDAC cohort. Shading represents 95% confidence intervals. (L) Western blot analysis of LAMB3 expression in MIA PaCa‐2 and PANC‐1 cells under normoxic or hypoxic (3% O_2_) conditions with different concentrations of 2‐DG treatment. (M) Western blot analysis of LAMB3 expression in MIA PaCa‐2 and PANC‐1 cells with or without HIF‐1α knockdown. Statistical analysis was performed using a two‐tailed Wilcoxon rank‐sum test (B, F, J, and K). For F, J, and K, data are shown as mean ± SD. ^*^ *p* < 0.05, ^**^ *p* < 0.01, ^***^ *p* < 0.001.

Next, we explored the transcriptional dynamics of ductal cells throughout carcinogenesis (Figure S9A–C). A nonnegative matrix factorization (NMF) algorithm was utilized to deconvolve the tumoral ductal cells from each PDAC sample in our scRNA‐seq dataset. Ribosomal activity, cellular responses to stress, interferon signaling, and glycolysis meta‐programs (MP) were identified across patients, highlighting conserved biological processes within PDAC ductal cells (Figure 8A). Notably, the glycolysis MP was absent in IPMN ductal cells, suggesting a metabolic reprogramming during IPMN evolution (Figure S9E). Coherently, the glycolysis score increased in a stage‐dependent manner, characterized by the progressive upregulation of key enzymes (*LDHA*, *HK2*) and transporters (*SLC2A1*) (Figure 8B,C). Consistently, gene set variation analysis (GSVA) of ductal cells across stages unveiled a marked activation of hypoxia and glycolysis pathways in the PDAC group (Figure S9D). These results highlight a significant and progressive glycolytic reprogramming in ductal cells during PDAC carcinogenesis, aligning with previous studies [51, 52].

We next evaluated the association between glycolytic activity and LAMB3 expression. Glycolysis scores in ductal cells were positively correlated with both *LAMB3* expression in ductal cells and LRRC15^+^ fibroblast features in the scRNA‐seq data (Figure 8D,E), implicating a potential role for glycolytic remodeling in modulating LAMB3 expression. This association was further supported by reanalysis of two independent bulk RNA‐seq datasets related to IPMN evolution (Figure S9F,G) [43, 44]. Concordant trends were observed at both mRNA and protein levels in PDAC samples from external datasets (Figures S9H and S10A–D). mIHC analysis revealed an accumulation of SLC2A1^+^ epithelial cells, which strongly correlated with increased LAMB3^+^ epithelial cell density during PDAC pathogenesis (Figure 8F–H). Notably, SLC2A1^+^LAMB3^+^ epithelial cells were highly enriched in PDAC tissues, yet nearly absent in uninvolved or IPMN tissues. Moreover, the density of LRRC15^+^ fibroblasts exhibited a positive correlation with the proportion of SLC2A1^+^LAMB3^+^ epithelial cells. mIHC analysis of an independent PDAC TMA cohort yielded similar findings. Patients with higher densities of SLC2A1^+^ epithelial cells showed higher densities of LAMB3^+^ epithelial cells, and conversely, higher LAMB3^+^ epithelial cell density was associated with increased SLC2A1^+^ epithelial cell proportions (Figure 8J). Notably, the abundance of SLC2A1^+^LAMB3^+^ epithelial cells showed a remarkable positive correlation with LRRC15^+^ fibroblasts, and their high density was associated with worse OS in PDAC (Figure 8I,K; Figure S10E,F).

Based on these findings, we hypothesized that enhanced glycolysis promotes LAMB3 induction. To test this under pathologically relevant conditions, we utilized hypoxic culture, a well‐characterized stimulus for glycolytic reprogramming in PDAC [53] Hypoxia markedly increased LAMB3 protein levels in both MIA PaCa‐2 and PANC‐1 cells (Figure 8L). To determine whether this upregulation depends on the increased glycolytic flux, cells were treated with increasing concentrations of 2‐deoxy‐D‐glucose (2‐DG) under hypoxia. Notably, 2‐DG treatment suppressed the hypoxia‐induced LAMB3 expression in a dose‐dependent manner, supports the notion that glycolytic reprogramming contributes to LAMB3 upregulation in PDAC cells. Given that HIF‐1α is a key mediator of hypoxia‐induced glycolytic reprogramming [54], we further examined its role in LAMB3 expression and found that HIF‐1α knockdown attenuated LAMB3 protein expression under hypoxic conditions (Figure 8M; Figure S10G,H). These findings demonstrate that hypoxia‐induced glycolytic reprogramming upregulates LAMB3 expression, at least in part through the HIF‐1α pathway.

## 3 Discussion

**FIGURE 9:**
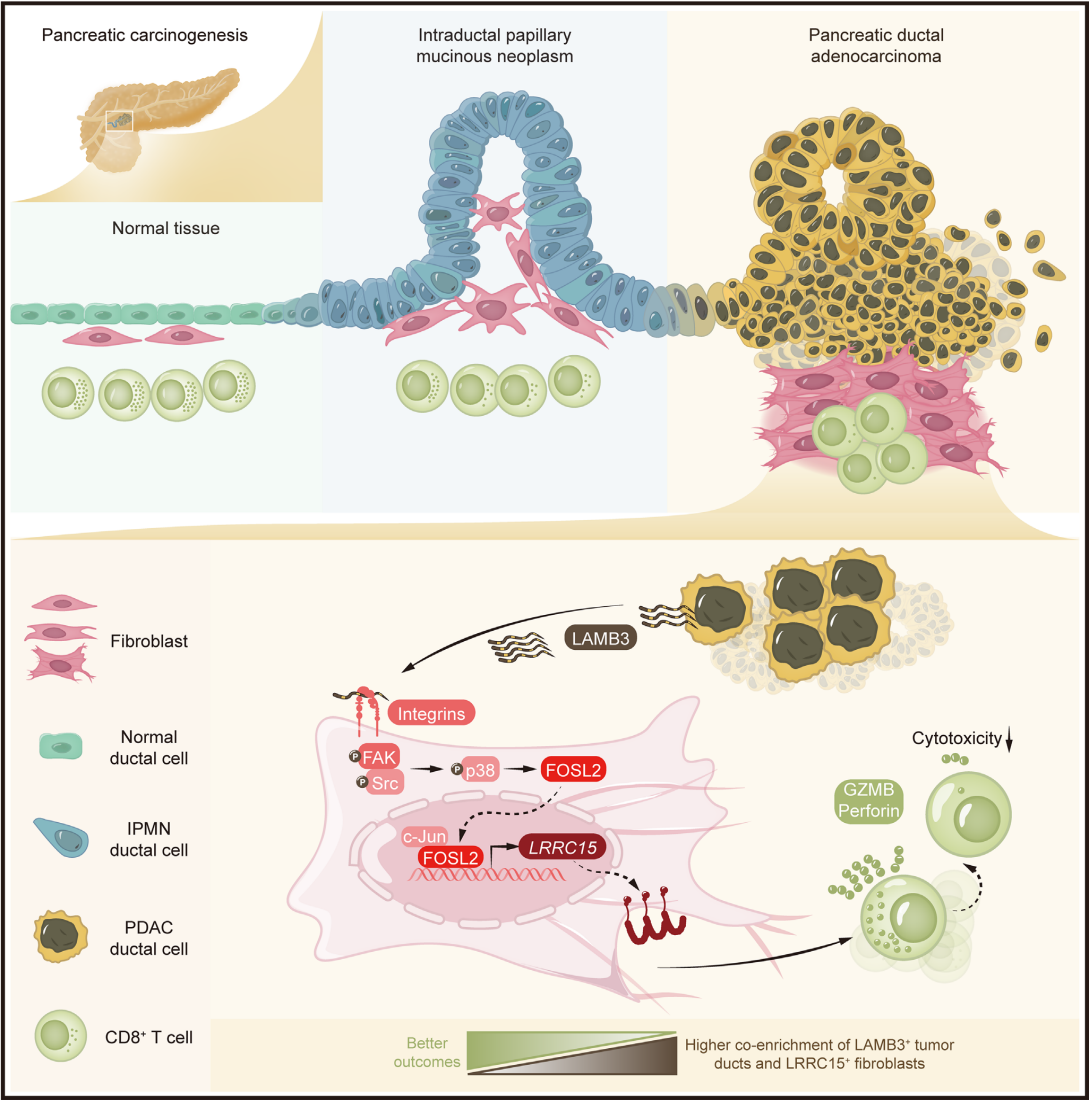
Graphical summary of major findings in this study.

An immunosuppressive TME represents a key barrier to effective immunotherapy in PDAC [2, 6, 55]. Understanding the co‐evolutionary dynamics among ductal cells, fibroblasts, and immune populations throughout PDAC development may yield critical mechanistic insights. In this study, we uncovered a progressive ductal‐fibroblast‐immune interaction network in which tumor ductal cell‐derived LAMB3 drives LRRC15^+^ fibroblast formation through the ITGB1/FAK/MAPK/FOSL2 pathway, ultimately contributing to immune evasion (Figure 9). Glycolytic reprogramming in PDAC ductal cells upregulated LAMB3 expression and was associated with increased LRRC15^+^ fibroblast enrichment. In orthotopic models, LAMB3 knockdown improved the efficacy of PD‐1 blockade. Clinically, the LAMB3‐LRRC15 axis correlated with poor overall survival. Together, these findings provide a mechanistic rationale for targeting the LAMB3‐LRRC15 axis as a strategy to overcome immunotherapy resistance in PDAC.

Previous studies have linked LRRC15^+^ fibroblasts to a myofibroblastic and immunosuppressive state [18, 19]. Consistently, our data also positioned LRRC15^+^ fibroblasts within the myofibroblast lineage in PDAC. Leveraging a multistage human PDAC development atlas, we further delineated their dynamic emergence during tumor evolution and demonstrated their association with CD8^+^ T cell dysfunction. In parallel, LRRC15‐OE CAFs impaired T cell cytolytic activity in vitro, further supporting a functional role for LRRC15^+^ fibroblasts in impairing T cell cytotoxicity. Together, these findings highlight a close coupling between stromal evolution and immune remodeling in human PDAC. We also gained mechanistic insight into the upstream regulation of LRRC15^+^ fibroblast formation. In contrast to the previously emphasized TGFβ‐driven mechanism of LRRC15^+^ fibroblast induction [18, 19], we identified PDAC ductal cell‐derived LAMB3 as an alternative upstream regulator of LRRC15^+^ fibroblast differentiation. Strikingly, rhLAMB3 robustly enhanced LRRC15^+^ fibroblast phenotype, supporting a role for LAMB3 in promoting LRRC15^+^ fibroblast formation through a TGFβ‐independent mechanism.

While previous studies have largely focused on the autocrine effects of LAMB3 in tumor cells, particularly its roles in promoting proliferation and invasion via PI3K‐AKT and EGFR cascades [26, 27, 28], our findings expand its function by revealing a complementary paracrine mechanism underlying fibroblast remodeling and immunosuppression. Specifically, tumor‐derived LAMB3 reshaped fibroblast plasticity, thereby indirectly compromising anti‐tumor immunity. Similar immunomodulatory roles of laminin family members have been reported in other malignancies, such as non‐small cell lung cancer and esophageal cancer, where LAMC2 facilitates T cell exclusion from tumor nests [56]. Importantly, our study further suggests that fibroblasts act as critical intermediary cells that translate tumor‐derived ECM signals into immunosuppressive programs.

Our data provide mechanistic insight into the LAMB3‐LRRC15 axis, supporting a model whereby tumor‐derived LAMB3 activates FAK/MAPK/FOSL2 signaling in fibroblasts and thereby promotes the formation of LRRC15^+^ fibroblasts. Mechanistically, we identified the AP‐1 family member FOSL2 as a transcriptional regulator of LRRC15 expression. Consistent with prior evidence demonstrating that AP‐1 family members, such as FOS, JUN, and NR4A1, govern fibroblast subset transitions [57], our data revealed elevated FOSL2 regulon activity in LRRC15^+^ fibroblasts compared to their negative counterparts. Dual‐luciferase reporter and ChIP analyses further supported direct transcriptional regulation of LRRC15 by FOSL2. Importantly, given the established role of FAK signaling in immune evasion across various solid tumors, including PDAC [58, 59], our study unveiled an additional mechanism by which FAK promotes immune suppression through driving the formation of LRRC15^+^ fibroblasts. Pharmacologic FAK inhibition attenuated these effects in orthotopic PDAC models, thereby extending our in vitro observations to an in vivo setting. Together, these findings identify the FAK/MAPK/FOSL2 cascade as a key mechanistic link connecting LAMB3 signaling to LRRC15^+^ fibroblast formation in PDAC.

Building on the elucidated crosstalk dynamics, we confirmed the clinical relevance of ductal‐fibroblast‐immune interaction through histopathological analyses across distinct clinical cohorts. We comprehensively profiled the expression pattern of LAMB3 throughout PDAC pathogenesis. LAMB3 exhibited a progressive upregulation in ductal cells during the initiation of IPMN and its progression to PDAC at both the mRNA and protein levels. This finding extends prior observations of elevated LAMB3 expression in PDAC tissues relative to normal pancreas, as well as its detectable presence in IPMN ductal cells [26, 60]. The predominant localization of LAMB3 expression within PDAC ductal cells rather than stromal compartments provides a rationale for its potential utility as a progression biomarker during IPMN surveillance, which warrants large‐scale validation through needle biopsy. While a previous study has associated diffuse high LAMB3 expression with adverse clinical outcomes in PDAC [26], our analysis identified tumor‐specific LAMB3 overexpression as an independent predictor of unfavorable OS. Importantly, concurrently high expression of both tumor‐specific LAMB3 and stroma‐specific LRRC15 confers poor prognosis, emphasizing the pro‐tumorigenic role and prognostic value of the LAMB3‐LRRC15 axis, which may inform risk‐stratification strategies. We also explored whether this axis might be associated with immunotherapy response. In a small pilot cohort treated with a non‐standard anti‐CD73 plus anti‐PD‐1 regimen, mIHC findings suggested a possible association between the LAMB3‐LRRC15 axis and treatment response. Importantly, the clinical association was functionally supported by orthotopic PDAC models. LAMB3 knockdown enhanced the anti‐tumor efficacy of PD‐1 blockade, accompanied by reduced LRRC15 positive area and restoration of CD8^+^ T cell cytotoxic function. Taken together, evidence from human cohorts and mouse models suggests that the LAMB3‐LRRC15 axis may represent a potential therapeutic vulnerability in PDAC, warranting further investigation in larger and more clinically representative cohorts.

In addition to elucidating the LAMB3‐LRRC15 axis, we identified upstream metabolic regulators of LAMB3 expression in PDAC ductal cells. Prior evidence has highlighted the role of metabolic pathways in ECM protein synthesis [61]. Glycolytic reprogramming, a major form of metabolic rewiring, has been widely implicated in tumor proliferation, metastasis, and immune escape [62]. However, the functional link between glycolysis and LAMB3 expression remains largely undefined, with only limited evidence suggesting a potential association in PDAC [63]. Through spatial co‐localization analyses and in vitro validation, we demonstrated that hypoxia‐induced glycolytic reprogramming upregulates LAMB3 expression in PDAC ductal cells through the HIF‐1α pathway. Notably, enhanced glycolytic activity in PDAC ductal cells was also associated with increased enrichment of LRRC15^+^ fibroblasts, linking glycolytic reprogramming to LAMB3‐associated fibroblast plasticity. Together, these findings provide insight into the metabolic regulation of LAMB3, and support further investigation into how glycolytic rewiring may influence stromal remodeling in PDAC.

In addition, our scRNA‐seq analysis suggested increased relative representation of Treg, impaired cytolytic function of CD8^+^ T cells, and enhanced tumor‐promoting macrophage states during PDAC development. These immune alterations consolidate the progressive establishment of an immunosuppressive niche, consistent with previous studies [9, 10, 11]. We also observed increased relative representation of MAIT cells during PDAC pathogenesis and highlighted their potential clinical relevance. Given the multifaceted roles of MAIT cells across various solid tumors [64, 65, 66], further interrogation into its function and regulatory mechanisms in PDAC is warranted.

Our study has several limitations. First, the scRNA‐seq dataset comprised a limited number of samples, which may constrain the generalizability of our findings. Second, the human immunotherapy cohort was a small pilot cohort treated with a non‐standard regimen, thereby limiting the generalizability of these findings to more commonly used immunotherapeutic settings. Third, although we identified molecular signatures associated with IPMN initiation and its progression to PDAC, functional validation was restricted to PDAC cell lines because of the absence of well‐characterized IPMN‐derived models. Future studies using organoids or genetically engineered mouse models are warranted. Fourth, bona fide LRRC15^+^ fibroblasts directly isolated from human PDAC tissues were not available for direct assessment of their effects on T cell cytotoxicity. In addition, the direct ligand‐receptor pairs or secreted factors responsible for the immunosuppressive effect of LRRC15^+^ fibroblasts on CD8^+^ T cells remain to be identified. Fifth, although CellChat analysis nominated LAMB3‐ITGB1 as a candidate ligand‐receptor interaction, and our perturbation experiments supported an ITGB1‐dependent downstream signaling axis mediating LAMB3‐induced effects in fibroblasts, further studies are required to directly validate this interaction and to determine whether LAMB3‐ITGB1 functions as a specific ligand‐receptor pair.

## 4 Conclusion

In conclusion, we delineate the coordinated evolution of ductal‐fibroblast‐immune crosstalk across the continuum from uninvolved pancreas through IPMN to PDAC, and elucidate the molecular mechanism of PDAC ductal cell‐secreted LAMB3‐mediated immune evasion by promoting LRRC15^+^ fibroblast formation. Our study implicates the LAMB3‐LRRC15 axis as a promising therapeutic target in PDAC.

## 5 Methods

This research was approved by the Ethics Committee of Peking Union Medical College Hospital (PUMCH) (I‐22PJ1025). Written informed consent was obtained from all participants. Histopathological diagnoses were independently confirmed by two experienced pathologists.

### 5.1 scRNA‐seq and Spatial Transcriptomics Cohort

Fresh samples were collected from 1 patient with IPMN and 8 patients with IPMN‐associated PDAC during surgery. Additionally, we incorporated 4 UNIN specimens from 4 donors sourced from published datasets. For spatial transcriptomics, additional fresh tissue samples were obtained from patients P04 and P05. Patient information is listed in Table S1.

### 5.2 TMA PDAC Cohort

In this cohort, PDAC patients undergoing surgery at PUMCH between January 2020 and February 2023 were consecutively enrolled. Patients lacking prognosis information or diagnosed with concurrent other malignancies were excluded, resulting in a final cohort of 160 cases. Two representative tumor regions were selected for each patient to construct TMA cores, with each core measuring 2 mm in diameter. In the IHC assay, 13 patients were excluded due to detachment of both cores. Similarly, 2 patients were excluded from mIHC analysis for the same reason. Tumor staging was determined according to the 8th edition of the American Joint Committee on Cancer TNM classification for PDAC. OS was assessed from the date of surgery to death or last follow‐up.

### 5.3 Immunotherapy Cohort

This exploratory cohort comprised 7 patients with PDAC treated at PUMCH. This was not a standard first‐line treatment cohort for PDAC. Specifically, all patients had previously undergone surgical resection and had also received neoadjuvant and/or adjuvant chemotherapy according to their clinical course. After postoperative recurrence and/or metastatic progression during follow‐up, patients were enrolled in a clinical trial and subsequently treated with combined anti‐CD73 and anti‐PD‐1 immunotherapy. Radiographic evaluations were initiated at approximately 6 weeks after treatment initiation and were repeated every 6 weeks thereafter. Treatment responses were assessed according to immune Response Evaluation Criteria in Solid Tumors (iRECIST) [67], and the best overall response was used as the endpoint for treatment efficacy. Among the 7 patients, 2 achieved immune partial response (iPR), 2 had immune stable disease (iSD), and 3 developed immune progressive disease (iPD). mIHC was performed on archival formalin‐fixed paraffin‐embedded (FFPE) specimens obtained from surgical resection, all of which had been collected prior to immunotherapy initiation. This cohort was analyzed as an exploratory clinical trial population of patients with postoperative recurrent and/or metastatic PDAC rather than a conventional standard‐of‐care first‐line treatment population. Detailed clinical characteristics are summarized in Table S2.

### 5.4 scRNA‐seq Data Generation and Quality Control

Fresh specimens were mechanically minced into small pieces and enzymatically digested. Following washing, cell counting, and concentration, single‐cell suspensions were prepared and subjected to microfluidic partitioning and barcoding. RNA from the barcoded cells was reverse‐transcribed, and sequencing libraries were constructed utilizing Chromium Single Cell 3’ v3 reagent kit (10x Genomics). All steps were performed following the standard manufacturer's protocols. FASTQ files were aligned to the GRCh38 human reference genome using Cell Ranger (v7.0.0). Downstream processing and analysis were conducted in R (v4.2.1). Low‐quality cells were filtered out based on the following criteria: fewer than 200 detected genes, fewer than 1000 unique molecular identifiers (UMI), or more than 15% mitochondrial genes. Suspected doublets were identified by DoubletFinder (v2.0.3) (https://github.com/chris‐mcginnis‐ucsf/DoubletFinder/) or by the co‐expression of marker genes from at least two major cell types, and were removed from downstream analysis.

### 5.5 scRNA‐seq Data Annotation

The remaining high‐quality cells underwent normalization and scaling with Seurat (v4.1.1) (https://satijalab.org/seurat/). We discarded batch effects across patients using Harmony (v0.1.0) (https://github.com/immunogenomics/harmony/) [68]. Major cell types were annotated according to canonical marker genes listed in Table S3. For subpopulation annotation, individual major cell types were extracted and re‐clustered. Subclusters were annotated based on the differentially expressed genes.

### 5.6 Spatial Transcriptomic Data Generation

Fresh tissue specimens were embedded in optimal cutting temperature compound and snap‐frozen in dry ice. Following tissue permeabilization optimization, spatial RNA expression profiling of cryosections was performed using the Visium Spatial Gene Expression Slide and Reagent Kit (10x Genomics) and subsequently sequenced. All steps were performed following the standard manufacturer's protocols. FASTQ files were aligned to the GRCh38 human reference genome using Space Ranger (v2.0.0). Histological annotations of spatial transcriptomics data were conducted by two experienced pathologists using Loupe Browser (v8.0.0). Spot‐level gene expression data were normalized using Seurat (v4.1.1).

### 5.7 Signature Scoring Analysis

Signature scoring for both scRNA‐seq and spatial transcriptomic data was conducted using the AddModuleScore function in the R package Seurat, based on gene sets listed in Table S4.

### 5.8 Pseudo‐Time Trajectory Analysis

To reconstruct the differentiation trajectories of fibroblasts, Monocle 2 toolkit (v2.28) (https://cole‐trapnell‐lab.github.io/monocle‐release/) was utilized [39]. After identification of 2000 highly variable genes, differentially expressed genes among fibroblast subpopulations with adjusted *p* < 0.05 were selected as ordering genes. The raw count matrix of these genes was extracted from the Seurat object and converted into a CellDataSet using newCellDataSet with expressionFamily = negbinomial.size() and lowerDetectionLimit = 0.5. Size factors and dispersions were estimated using estimateSizeFactors and estimateDispersions, followed by gene detection using detectGenes with min_expr = 0.1. Ordering genes were defined using setOrderingFilter. Dimensionality reduction was performed using reduceDimension with reduction_method = DDRTree, max_components = 2, norm_method = log, and residualModelFormulaStr = “∼num_genes_expressed”, and cells were ordered in pseudo‐time using orderCells with root_state = 4. Branch‐dependent transcriptional changes were analyzed using BEAM, and pseudo‐time‐dependent genes were identified using differentialGeneTest.

### 5.9 Transcription Factor Analysis

To compare regulon activity between LRRC15 positive fibroblasts and their negative counterparts, the SCENIC (v3) (https://github.com/aertslab/SCENIC/) algorithm was implemented [45]. Seurat objects of all LRRC15 positive fibroblasts and a random subset of 1000 LRRC15 negative fibroblasts were imputed. SCENIC analysis was conducted with default parameters.

### 5.10 Cell‐Cell Interaction Analysis

To decipher the cell‐cell communication of fibroblasts, CellChat (v1.6.1) (https://github.com/sqjin/CellChat/) was implemented to infer intercellular signaling networks [42]. Matrices of total cells were split based on major cell type annotations across sample stages to construct stage‐specific CellChat objects. After predicting interaction networks for each stage, we compared the communication probabilities of all signaling pathways received by fibroblasts across stages. Additionally, we compared the communication probabilities of the ligand‐receptor pairs targeting fibroblasts, with particular attention to signaling inputs originating from ductal cells.

### 5.11 Meta‐Programs Analysis

To explore the intra‐tumor heterogeneity, the non‐negative factorization algorithm in the R NMF package (v0.25) was implemented in ductal cells from PDAC and IPMN samples according to prior studies [69]. Expression matrices were normalized and scaled, and genes whose expression standard deviation was less than 0.5 were excluded for NMF analysis. Five programs and their top 50 genes were extracted per sample. To explore shared intra‐tumor programs across samples, Jaccard similarity analysis was applied to quantify overlapping genes in each program. Meta‐programs were identified by clustering programs based on Jaccard similarity.

### 5.12 GSVA Pathway Analysis

To investigate the dynamic changes in cancer hallmark pathways within ductal cells during PDAC pathogenesis, R package GSVA (v1.46.0) was applied. Hallmark gene sets were retrieved from the “msigdbr” database (http://software.broadinstitute.org/gsea/msigdb/) [70]. And GSVA scores were then z‐scored to facilitate visualization.

### 5.13 Spatial Transcriptomic Proximity Analysis

To assess the spatial correlation between *LAMB3* expression and LRRC15^pos^fibroblast signature, R package SPATA2 (v2.0.4) (https://themilolab.github.io/SPATA2/) and SPATAwrappers (v0.0.9) (https://github.com/heilandd/SPATAwrappers/) were applied following previously published studies [71, 72]. First, spot‐wise *LAMB3* expression, LRRC15^pos^fibroblast signature, and their tissue coordinates were extracted from the Seurat object to generate SPATA objects. Function inferJuxtaposition from SPATAwrappers package was utilized to predict the likelihood of spatial proximity between the two features. Function plotJuxtaposition in the same package was then employed to visualize the results.

### 5.14 Bulk RNA‐seq Analysis for Public Datasets

To analyze gene expression dynamics during IPMN evolution, two publicly available bulk RNA‐seq datasets were utilized. The first dataset from Liffers et al., included normal acinar tissue (n = 3), LGD‐IPMN (n = 12), HGD‐IPMN (n = 12), and cPDAC (n = 4) samples [44]. The second dataset from Hiraoka et al., comprised normal tissue (n = 7), LGD‐IPMN (n = 6), HGD‐IPMN (n = 6), and PDAC (n = 3) samples [43]. To analyze expression correlations in PDAC, bulk RNA‐seq from 150 samples was obtained from the TCGA‐PAAD cohort (https://tcga‐data.nci.nih.gov/tcga/) [73]. After filtering lowly expressed genes across samples, scoring of glycolysis and LRRC15^pos^fibroblast signature was performed using R package singscore (v1.18.0) (https://github.com/DavisLaboratory/singscore/) [74]. Expression levels of individual genes were represented as log2 (TPM+1).

### 5.15 Proteome Analysis for Public Datasets

To compare the protein‐level expression difference between normal pancreatic tissue and PDAC tissue, proteomic data from 44 normal pancreatic and 105 PDAC tissue samples were downloaded from the CPTAC Pan‐Cancer Data portal (https://pdc.cancer.gov/pdc/cptac‐pancancer/) [38]. After transforming protein expression into log2 value and filling the missing values using R package NAguideR (v0.2.0) (https://github.com/wangshisheng/NAguideR/), we scored glycolysis and LRRC15^pos^fibroblast signature using R package singscore.

### 5.16 IHC Staining and Analysis

FFPE sections from the human IPMN evolution cohort, PDAC TMA cohort, and mouse orthotopic tumors were subjected to IHC analysis. FFPE sections were deparaffinized, rehydrated, and subjected to antigen retrieval, followed by blocking of endogenous peroxidase activity and nonspecific binding sites. Sections were incubated overnight at 4°C with primary antibodies against LAMB3 (1:50, Santa Cruz, Cat. No. sc‐133178) or LRRC15 (human samples: 1:400, Abcam, Cat. No. ab150376, UK; mouse samples: 1:1000, Cell Signaling Technology, Cat. No. 50546). After incubation with secondary antibody, sections were stained with DAB substrate and counterstained with hematoxylin. The stained sections were scanned for further analysis. For human samples, two experienced pathologists assessed H‐scores using QuPath (v0.5.1). Staining intensity for each marker was classified into 4 grades: 0 (negative staining), 1 (weak staining), 2 (moderate staining), and 3 (strong staining). H‐scores were calculated according to the percentage of each staining intensity within the same lesion from a single patient. For mouse tumors, the proportion of LRRC15 positive area was quantified.

### 5.17 mIHC Staining and Analysis

mIHC staining was performed on FFPE sections from the IPMN cohort, TMA PDAC cohort, and orthotopic tumor samples using an Opal Polaris 7‐color Kit (Akoya Biosciences, USA). All steps were performed following the manufacturer's standard protocols. Briefly, sections were deparaffinized in xylene, rehydrated in graded ethanol, and subjected to heat‐mediated antigen retrieval in AR6 buffer. Sections were then incubated with primary antibodies, secondary‐HRP antibodies, and opal tyramide signal amplification (TSA) dyes in each round. The antibodies and corresponding opals used were shown in Table S5. After counterstaining with DAPI, sections were scanned using the Vectra multispectral slide scanner (Vectra 3.0, PerkinElmer), and representative images were exported. Images of each section were imported into QuPath for cell detection and positive cell calculation [75]. For the IPMN cohort, circle regions of interest (ROI) with a diameter of 1 mm were randomly selected. For the TMA PDAC cohort, TMA dearrayer and tissue segmentation functions were conducted to classify each core prior to cell detection.

### 5.18 Survival Analysis

For in‐house survival analyses, IHC H‐scores from 147 patients in the PDAC tissue microarray (TMA) cohort and mIHC data from 158 patients in the same cohort were analyzed. For public survival analysis, 150 PDAC samples from the TCGA‐PAAD cohort and 97 PDAC samples from the CPTAC‐PDAC cohort were included. Patients were classified into 2 groups based on the median value of each parameter. Survival analysis was performed employing the survfit function in the R package survival (v3.5.5). Univariate and multivariate Cox regression analyses were conducted using the coxph function from the same package. Forest plots were visualized using the R package forestploter (v1.1.3).

### 5.19 Isolation and Culturing of Primary CAFs From PDAC

Primary CAFs were isolated from surgically resected fresh PDAC tissues using the outgrowth approach [15]. Isolated CAFs were cultured in Dulbecco's Modified Eagle's Medium (DMEM)/F‐12 (1:1) (Gibco, Cat. No. 11320033) supplemented with 10% fetal bovine serum (FBS) (Corning, Cat. No. 35‐010‐CV, New York, USA) in a 37°C humidified atmosphere with 95% air and 5% CO_2_. Passage numbers 2–8 were used for the assays.

### 5.20 Isolation and Culture of Primary Human CD8^+^ T Cells

Peripheral blood was collected from healthy donors. Within 1 h of collection, peripheral blood mononuclear cells (PBMCs) were isolated using SepMate‐50 tubes (STEMCELL, Cat. No. 85450) in combination with Lymphoprep (STEMCELL, Cat. No. 07851), followed by centrifugation at 1200 × g for 10 min. CD8^+^ T cells were then isolated from PBMCs using the Human CD8^+^ T Cell Isolation Kit (STEMCELL, Cat. No. 17953) and an EasySep magnet (STEMCELL, Cat. No. 18000). Purified CD8^+^ T cells were cultured in ImmunoCult‐XF T Cell Expansion Medium (STEMCELL, Cat. No. 10981) supplemented with 20 U/mL Human IL‐2 Recombinant Protein (PeproTech, Cat. No. 102312). For activation, CD8^+^ T cells were stimulated with Dynabeads T‐activator CD3/CD28 beads (Gibco, Cat. No. 11131D), and the beads were subsequently removed using a magnet (Invitrogen, Cat. No. 12321D) after 72 h. All procedures were performed according to the manufacturers’ instructions.

### 5.21 Culturing of Human Cell Lines

Human cell lines AsPC‐1 (RRID:CVCL_0152), BxPC‐3 (RRID:CVCL_0186), MIA PaCa‐2 (RRID:CVCL_0428), PANC‐1 (RRID:CVCL_0480), HPNE (RRID:CVCL_C466), and HEK293T (RRID:CVCL_0063) were obtained from the American Type Culture Collection (ATCC) in 2018. The mouse KPC cell line, is a KPC mouse model‐derived PDAC cell line, was a gift from Prof. Taiping Zhang (PUMCH, Beijing, China) [76]. MIA PaCa‐2, PANC‐1, and HEK293T cells were cultured in DMEM (Gibco, Cat. No. C11965500BT) supplemented with 10% FBS. AsPC‐1 and BxPC‐3 were kept in RPMI Medium 1640 basic (Gibco, Thermo Fisher Scientific, USA) and 10% FBS. HPNE were cultured in a mixed medium containing 75% DMEM with low glucose (Sigma, Cat. No. D5030‐10 × 1 L, USA) and 25% Medium M3 Base (Incell corp, Cat. No. M300F‐500, USA), supplemented with the following components: 10% FBS, 5.5 mM D‐glucose (Sigma–Aldrich, Cat. No. G7021‐100G), 10 ng/mL human recombinant EGF (PeProtech, Cat. No. AF‐100‐15; USA), 1 × GlutaMaxTM Supplement (Gibco, Cat. No. 35050061), and 750 ng/mL puromycin (Gibco, Cat. No. A1113803). KPC cells were cultured in DMEM with 10% FBS, and 2 mm glutamine (Gibco, Cat. No. 25030081). All cell lines were authenticated by short tandem repeat profiling. No evidence of mycoplasma contamination was observed during routine culture.

### 5.22 Overexpression and siRNA Transfection

Plasmids designed to overexpress LAMB3, FOSL2, or LRRC15 were purchased from Genechem Company (Shanghai, China) and transfected into PDAC cells (MIA PaCa‐2 and PANC‐1) or CAFs using Lipofectamine 3000 (Invitrogen, Thermo Fisher Scientific, Cat. No. 3000155) at 70%–80% confluence. siRNAs targeting ITGB1 (Hanbio, China), FOSL2, and HIF‐1α (RiboBio, China) were transfected into CAFs or PDAC cells using Lipofectamine siRNA iMAX (Invitrogen, Thermo Fisher Scientific, Cat. No. 13778) at 60%–70% confluence. The interference sequences used are provided in Table S6.

### 5.23 Co‐Culture CAFs With PDAC Cell‐Conditioned Medium

CAFs were seeded in plates and cultured for 48 h in a 1:1 mixture of conditioned medium derived from PDAC cells (MIA PaCa‐2, PANC‐1) and fresh CAFs growth medium.

### 5.24 Co‐Culture of Activated Primary Human CD8^+^ T Cells With CAFs

Following treatment of CAFs with either LRRC15‐OE plasmid, rhLAMB3 (FineTest, Cat. No. P8961, China), or conditioned medium from LAMB3‐OE PDAC cells (MIA PaCa‐2, PANC‐1), CD8^+^ T cells were co‐cultured with CAFs for 24 h. The co‐culture was conducted at a CAF‐to‐CD8^+^ T cell ratio of 1:1 using a 1:1 mixture of the respective growth medium.

### 5.25 Generation of Stable LAMB3‐Knockdown and LAMB3‐Overexpressing KPC Cells

Stable LAMB3‐knockdown and LAMB3‐overexpressing KPC cells were generated using lentiviral transduction. Lentiviruses encoding shRNAs targeting LAMB3 or the full‐length LAMB3 coding sequence, together with their corresponding control vectors, were purchased from GeneChem (WZ Bioscience, Shandong, China). Cells were infected with lentiviruses when they reached 20%–30% confluence and subsequently selected with puromycin for 2 weeks to establish stable cell lines. Knockdown or overexpression efficiency was confirmed by Western blot analysis. The sequences used were provided in Table S7.

### 5.26 Orthotopic PDAC Models and In Vivo Therapeutic Interventions

All animal experiments were approved by the Animal Welfare & Ethics Committee of Peking Union Medical College Hospital (PUMCH) (Approval No. XHDW‐2025‐220) and performed in accordance with institutional guidelines. Female C57BL/6 mice aged 6–8 weeks were used. For orthotopic implantation, 2 × 10^5^ KPC cells in 20 µL PBS were mixed with 20 µL Matrigel and injected into the pancreas of each mouse. After successful tumor implantation confirmed by in vivo bioluminescence imaging, mice were randomly assigned to four groups. For FAK inhibition experiments, mice received VS‐4718 (50 mg/kg), formulated in vehicle (0.5% carboxymethyl cellulose and 0.1% Tween‐80 in sterile water), by oral gavage twice daily from day 6 after implantation until the experimental endpoint. Control mice received the same vehicle volume alone. For anti‐PD‐1 treatment experiments, mice received anti‐PD‐1 antibody (clone RMP1‐14, BioXcell) (200 µg per treatment) by intraperitoneal injection starting on day 10 after implantation, three times per week for 2 weeks. Mice were euthanized on day 21 after implantation, and tumors were collected for subsequent analyses.

### 5.27 Dissociation of Mouse Tumors

Tumors were minced into small fragments. Single‐cell suspensions were prepared using the Mouse Tumor Dissociation Kit (Miltenyi Biotec, Cat. No. 130‐095‐929) and gentleMACS Octo Dissociator under modified conditions optimized for preservation of tumor‐infiltrating lymphocytes. Briefly, tissues were digested in DMEM containing the enzyme, using the predefined program 37C_m_TDK_1. The dissociated samples were filtered through a 70 µm cell strainer, washed with serum‐containing medium, and centrifuged at 300 × g for 7 min at 4°C. Cell pellets were then used for subsequent flow cytometric analysis.

### 5.28 Flow Cytometry

Activated primary human CD8^+^ T cells were harvested after co‐culture with CAFs and restimulated for 6 h using Cell Activation Cocktail containing Brefeldin A (BioLegend, Cat. No. 423303). Cells were then stained with FITC anti‐human CD8 antibody (BioLegend, Cat. No. 344703). After surface staining, cells were fixed, permeabilized, and stained intracellularly with the following antibodies: APC anti‐human/mouse Granzyme B Recombinant (BioLegend, Cat. No. 372203), PE/Cyanine7 anti‐human IFN‐γ (BioLegend, Cat. No. 502527), and PE anti‐human Perforin (BioLegend, Cat. No. 353303). For flow cytometric analysis of mouse tumor single‐cell suspensions, cells were stained with Zombie NIR (BioLegend, Cat. No. 423105), Alexa Fluor 700 anti‐mouse CD45 (BioLegend, Cat. No. 147715), PE/Cyanine7 anti‐mouse CD8a (BioLegend, Cat. No. 100721), and APC anti‐human/mouse Granzyme B Recombinant (BioLegend, Cat. No. 372203). Flow cytometry was performed on a BD LSRFortessa platform, and data were analyzed using FlowJo software (v10.10).

### 5.29 Western Blot

Total protein was extracted using RIPA lysis buffer supplemented with protease inhibitor and phosphatase inhibitor cocktails (Roche Applied Science). Protein concentrations were determined using Pierce BCA Protein Assay Kit (Thermo Fisher Scientific, USA). Protein lysates were separated by SDS‐PAGE and transferred onto polyvinylidene difluoride (PVDF) membranes (Millipore, Billerica, USA). After blocking with 5% non‐fat milk, membranes were incubated overnight at 4°C with primary antibodies, followed by incubation with appropriate secondary antibodies. Protein bands were visualized using the ChemiDoc Touch Imaging System (Bio‐Rad). GAPDH or β‐actin served as a reference control. All antibodies used are listed in Table S8.

### 5.30 ELISA Assay

Levels of LAMB3 protein in conditioned media from CAFs and PDAC cells were quantified using a Human LAMB3 ELISA Kit (FineTest, Cat. No. EH1641, China) following the manufacturer's instructions. Briefly, cells were cultured in serum‐free media until reaching 80% confluence. Supernatants were collected and incubated in ELISA plates at 37°C for 1.5 h, and the plates were subsequently incubated with the detection antibody, HRP‐SABC, and TMB substrate. Absorbance was measured at 450 nm to determine relative protein concentration.

### 5.31 Quantitative Real‐Time PCR (qRT‐PCR) Assay

Total RNA was extracted from CAFs with or without rhLAMB3 treatment, CAFs with or without LRRC15‐OE manipulation using RNA‐Quick Purification Kit (Yishan Biotechnology Co., Ltd, Cat. No. RNOOl, China). cDNA was synthesized from the purified RNA using Prime Script RT Master Mix (TaKaRa, Japan). qRT‐PCR was performed using PowerUp SYBR Green Master Mix on the QuantStudio5 Real‐Time PCR System (Applied Biosystems, Thermo Fisher Scientific). GAPDH acted as the reference gene. Primer sequences are presented in Table S9.

### 5.32 Luciferase Reporter Assay

LRRC15 reporter plasmids, FOSL2‐OE plasmids, and pRL‐TK were co‐transfected into HEK293T cells using lipofectamine 3000. After transfection, cells were seeded into 96‐well plates and cultured for 48 h. Luciferase and Renilla activities were detected using Dual–Luciferase Reporter Assay System (Promega, Cat. No. E1960, USA) according to the manufacturer's instructions.

### 5.33 ChIP‐qPCR Assay

ChIP was conducted using SimpleChIP Plus Enzymatic Chromatin IP Kit (Cell Signaling Technology, Cat. No. 9005) following the manufacturer's instructions. Immunoprecipitated DNA was analyzed by qRT‐PCR. Primers for ChIP‐qPCR are listed in Table S10.

### 5.34 Statistics

Statistical analyses were performed using GraphPad Prism 9 (GraphPad Software Inc., San Diego, CA, USA) and R software (v4.2.1). Data are presented as mean ± SD unless otherwise specified. Differences between two groups were assessed using an unpaired two‐tailed Student's *t*‐test or two‐tailed Wilcoxon rank‐sum test, and comparisons among multiple groups were performed using Tukey's post hoc test. Survival analyses were conducted using the Kaplan–Meier method, and differences between groups were evaluated using the log‐rank test. Pairwise comparisons were performed for multi‐group survival analyses. Univariate and multivariate Cox proportional hazards regression models were used to identify independent prognostic factors. Correlation analyses were performed using Pearson's correlation or Spearman's rank correlation. A two‐sided *p* value < 0.05 was considered statistically significant (^*^ *p* < 0.05, ^**^ *p* < 0.01, ^***^ *p* < 0.001).

## Author Contributions

Z.L., H.W., F.B., and M.D. conceived the study and supervised the overall research. X.S. enrolled patients, collected clinical data, performed bioinformatics analyses, and conducted IHC experiments. H.L. and J.S. contributed to in vitro experiments. X.S., H.L., and J.S. contributed to in vivo experiments. X.L., R.L., J.L., and H.Z. conducted histopathological evaluations. X.X. assisted with bioinformatics analysis. X.J. and Y.W. assisted with experiments. S.L., J.P., and Y.C. assisted with histological assays. L.C., Y.Z., X.K., J.W., X.Y., B.L., and Q.L. contributed to TMA cohort construction. X.S. drafted the manuscript. H.L., F.B., H.W., and Z.L. reviewed and revised the manuscript. All authors provided feedback.

## Funding

This work was supported by the Capital's Funds for Health Improvement and Research (CFH) (2024‐2‐4012). The National High Level Hospital Clinical Research Funding (2025‐PUMCH‐D‐002), the National Key Research and Development Program of China (2025YFC3410200), the National Science Fund for Distinguished Young Scholars (T2125002), the Noncommunicable Chronic Diseases‐National Science and Technology Major Project (2025ZD0544800), the National High Level Hospital Clinical Research Funding (2022‐PUMCH‐B‐061), the Chinese Academy of Medical Sciences (CAMS) Innovation Fund for Medical Sciences (CIFMS) (2023‐I2M‐C&T‐B‐022), the Beijing Municipal Natural Science Foundation (L252174), and the National Natural Science Foundation of China (82403474) also provided funding to conduct this project.

## Conflicts of Interest

The authors declare no conflicts of interest.

## Supporting information




**Supporting File 1**: advs75719‐sup‐0001‐SuppMat.docx.

**Supporting File 2**: advs75719‐sup‐0002‐TableS11.xlsx.

## Data Availability

The raw scRNA‐seq data generated in this study, together with relevant sample metadata, have been deposited in the Genome Sequence Archive for Human (GSA‐Human) repository under accession number HRA015868 (https://ngdc.cncb.ac.cn/gsa‐human/browse/HRA015868). Previously published data used in this study are available from the GEO datasets (https://www.ncbi.nlm.nih.gov/geo/) under accession codes GSE155698, GSE210351, and GSE19650. De‐identified processed count data from the scRNA‐seq dataset and other data supporting the findings of this study are available from the corresponding author upon reasonable request. To facilitate data interpretation and reuse, the cell annotation table used in this study is provided as Table S11.
