# Supplementary material for: Tumor‐Derived LAMB3 Drives Immunosuppressive LRRC15+ Fibroblast Formation During Pancreatic Ductal Adenocarcinoma Development
Source: Adv Sci (Weinh). 2026 May 19;13(45):e20029. doi: 10.1002/advs.202520029 (PMC13336103; doi:10.1002/advs.202520029)
Supplement: Supplementary file 1 [file ADVS-13-e20029-s001.docx]

Supporting Information

Title: Tumor-derived LAMB3 drives immunosuppressive LRRC15^+^ fibroblast formation during pancreatic ductal adenocarcinoma development

**Author(s), and Corresponding Author(s)***

Xuqing Shi^1,6^, Hangqi Liu^1,6^, Jianru Sun^1^, Xiaoding Liu^1^, Xinping Jv^1^, Longyun Chen^1^, Yuhan Zhang^1^, Hui Zhang^1^, Xudong Xing^2^, Ruiyu Li^1^, Xinyi Ke^1^, Jun Wang^1^, Xianglin Yin^1^, Bohan Liu^1^, Qixian Liu^1^, Yuan Wang^1^, Junliang Lu^1^, Shiyi Liu^1^, Junyi Pang^1^, Yumeng Cai^1^, Menghua Dai^3,^*, Fan Bai^4,5,^*, Huanwen Wu^1,^* and Zhiyong Liang^1,^*

^1^Department of Pathology, State Key Laboratory of Common Mechanism Research for Major Diseases, Peking Union Medical College Hospital, Chinese Academy of Medical Sciences & Peking Union Medical College, Beijing, China.

^2^Beijing Institute of Genomics, Chinese Academy of Sciences and China National Center for Bioinformation, Beijing, China.

^3^Department of General Surgery, Peking Union Medical College Hospital, Chinese Academy of Medical Sciences & Peking Union Medical College, Beijing, China.

^4^Biomedical Pioneering Innovation Center (BIOPIC), Peking-Tsinghua Center for Life Sciences, State Key Laboratory of Metabolic Dysregulation & Prevention and Treatment of Esophageal Cancer, School of Life Sciences, Peking University (PKU), Beijing, China.

^5^Peking University Beijing-Tianjin-Hebei Biomedical Pioneering Innovation Center, Tianjin, China.

^6^These authors contributed equally (as first authors): Xuqing Shi, Hangqi Liu.

*These authors jointly supervised this work: Zhiyong Liang, Huanwen Wu, Fan Bai, and Menghua Dai.

E-mail: liangzy@pumch.cn (Z.L.), wuhuanwen10700@pumch.cn (H.W.), fbai@pku.edu.cn (F.B.), and daimh@pumch.cn (M.D.).

**Figure S1 to S10**

**Table S1 to S10**

**Figure S1. Overview of scRNA-seq analysis across 82,760 cells from UNIN, IPMN, and PDAC tissues. Related to Figure 1.** (**A**) Violin plots showing nCount_RNA, nFeature_RNA and percentage of mt_RNA in each sample. (**B**) Bar plots showing the numbers and relative proportions of recovered major cell types in each sample from the integrated scRNA-seq dataset. (**C**) Charts illustrating the composition of profiled cells across disease stages in the integrated scRNA-seq dataset. (**D**) UMAP plot visualizing the sample origin of total cells. (**E**) UMAP plots illustrating the distribution of major cell types across stages.The legends are shared with **B**. (**F**) Heatmap showing the top differentially expressed genes for each major cell type.

**Figure S2.** **Transcriptional characteristics of immune cells across disease stages. Related to Figure 1.** (**A**) UMAP visualization of distinct subsets of CD4^+^ T cells (left), CD8^+^ T cells (middle), and myeloid cells (right). (**B**) Violin plots displaying the functional scores of CD4^+^ T cell subsets. (**C**) Violin plots displaying the functional scores of CD8^+^ T cell subsets. (**D**) Violin plots comparing naive and exhaustion scores of total CD4^+^ T cells across stages (UNIN: n = 701; IPMN: n = 9,845; PDAC: n = 5,284). (**E**) Dot plot illustrating expression of representative exhaustion-related genes in CD4^+^ T cells across disease stages. (**F**) Heatmap showing the expression of selected genes from various signatures in total CD8^+^ T cells across stages. (**G**) Kaplan-Meier survival curve comparing the pan-cancer MAIT signature^high^ (n = 75) and pan-cancer MAIT signature^low^ (n = 75) patients in the TCGA-PAAD cohort. Genes included in the pan-cancer MAIT signature are listed. (**H**) Dot plot showing the expression of selected genes from LYVE^high^ TRM signature in macrophage subsets. (**I**) Violin plots showing functional scores of macrophages across stages (UNIN: n = 1,055; IPMN: n = 2,454; PDAC: n = 2,268). (**J**) Violin plots showing functional scores of macrophage subsets (Mac_SPP1: n = 4,180; Mac_FOLR2: n = 1,597). (**K**) Heatmap showing the expression of selected genes from various signatures in macrophages across stages. Statistical analysis was performed using two-tailed Wilcoxon rank-sum test (**D**, **I**, and **J**), and log-rank test (**G**).

**Figure S3.** **Transcriptional characteristics of fibroblasts across disease stages.** **Related to Figure 2 and 3.** (**A**) UMAP plots illustrating the distribution of fibroblast subsets across stages. (**B**) Heatmap depicting the top differentially expressed genes and enriched pathways for each subpopulation. Gene Ontology biological process terms were used for pathway annotation. (**C**) Heatmap showing the expression of signature genes representing different fibroblast taxonomies across fibroblast subpopulations. (**D**) UMAP plot showing the expression pattern of *LRRC15*. (**E**) Violin plots comparing functional scores of fibroblasts **across stages** (UNIN: n = 611; IPMN: n = 1,899; PDAC: n = 3,086). (**F**) Box plots showing LRRC15^pos^fibroblast signature scores and LRRC15 expression levels in the CPTAC-PAAD cohort (Normal: n = 44; Tumor: n = 105). (**G**) Scatter plots showing the Spearman correlation between the relative proportion of Fib_C7_IGF1high and Fib_LRRC15 among total fibroblasts across samples in the scRNA-seq dataset. Shading represents 95% confidence intervals. (**H**) Scatter plot depicting the Pearson correlation between *LRRC15* and *C7* expression levels in each fibroblast. (**I**) Western blot analysis of LRRC15 expression in CAFs with or without LRRC15 overexpression. Statistical analysis was performed using two-tailed Wilcoxon rank-sum test (**E** and **F**).

**Figure S4. LAMB3-mediated crosstalk is associated with the formation of LRRC15^+^ fibroblasts. Related to Figure 4.** (**A**) Bar plots comparing the total information flow from all major cell types to fibroblasts across different groups. (**B**) Dot plot revealing LAMININ signaling intensity from various major cell types to fibroblasts across different stages. Dot color and size represent the calculated communication probability and p-value, respectively. (**C**) Scatter plots showing **differentially expressed genes** in IPMN and PDAC ductal cells compared with **ductal cells in** uninvolved tissue. (**D**) Scatter plots showing the Spearman correlation between average *LAMB3* level in ductal cells and average *ITGB1* level in fibroblasts across each sample. Shading represents 95% confidence intervals. (**E**) Box plots showing *LAMB3* RNA level in epithelial cells across stages in two independent cohorts (left and middle) and **LAMB3 protein levels** in the CPTAC-PDAC cohort (right) (left, UNIN: n = 7; LGD: n = 6; HGD: n = 6; PDAC: n = 3) (middle, UNIN: n = 3; LGD: n = 12; HGD: n = 12; cPDAC: n = 4) (right, Normal: n = 44; Tumor: n = 105). (**F**) UMAP plot showing the **distribution of *LAMB3* expression**. (**G**) Scatter plots showing the Spearman correlations between *LAMB3* expression in ductal cells and fibroblast features across samples in the scRNA-seq dataset. Shading represents the 95% confidence interval. Statistical analysis was performed using two-tailed Wilcoxon rank-sum test (**E**).

**Figure S5. Association between LAMB3 expression and LRRC15^+^ fibroblasts across disease stages. Related to Figure 4.** (**A**) Scatter plots showing the Pearson correlation between protein levels of LAMB3 and features of LRRC15^+^ fibroblasts across samples in the CPTAC-PDAC cohort. Shading represents 95% confidence intervals. (**B**) Line diagram showing the spatial distance between LRRC15^pos^fibroblast signature and *LAMB3* expression in the P04 sample. The x-axis represents distance from spots with high LRRC15^pos^fibroblast signature and the y-axis represents *LAMB3* expression probability. (**C**) H&E staining (left) and pathological annotation (right) **of P05 sample**. (**D**) Spatial feature plots revealing the distribution of *LAMB3* expression and LRRC15^pos^fibroblast signature in P05 sample. (**E**) H&E staining (left) and pathological annotation (right) of P04 sample are displayed. (**F**) Spatial feature plots revealing the distribution of *LAMB3* expression and LRRC15^pos^fibroblast signature in P04 sample. (**G**) Representative mIHC images showing LRRC15^+^ fibroblasts in close proximity to LAMB3^+^ PDAC ductal cells from #Patient2. DAPI, LRRC15, PDPN, LAMB3, and PanCK are shown in blue, pink, red, yellow, and cyan, respectively. Scale bars, 25 μm.

**Figure S6. Clinical implications of co-expression of tumor-derived LAMB3 and LRRC15^+^ fibroblasts in PDAC. Related to Figure 5.** (**A**) Representative mIHC images from a patient with concurrently low tumor_LAMB3 and stroma_LRRC15 H-scores, as shown in **Figure 5C**. DAPI, LRRC15, PDPN, LAMB3, and PanCK are shown in blue, pink, red, yellow, and cyan, respectively. Scale bars: low magnification, 100 μm; high magnification, 25 μm. (**B**) Univariate Cox regression analysis identifying potential predictors of overall survival. (**C**) Multivariate Cox regression analysis evaluating the prognostic value of tumor_LAMB3 H-score groups alongside additional factors. (**D**) Multivariate Cox regression analyses evaluating the prognostic value of combined tumor_LAMB3 and stroma_LRRC15 groups together with other factors potentially predictive of overall survival. *P* values < 0.05 are shown in bold. (**E**) Kaplan-Meier survival curves showing the overall survival probability based on LAMB3 expression (left) or LRRC15^pos^Fib signature (right) in the CPTAC-PDAC cohort. (**F**) Kaplan-Meier survival curves showing overall survival stratified by combined LAMB3 expression and LRRC15^pos^Fib signature in the CPTAC-PDAC cohort. Statistical analysis was performed using log-rank test (**E** and **F**) and Tarone-Ware test (**F**).

**Figure S7. Associations between the ITGB1/FAK/MAPK/FOSL2 pathway in fibroblasts and LAMB3^+^ ductal cells, as well as LRRC15^+^ fibroblasts across disease stages. Related to Figure 6.** (**A**) RT-qPCR analysis of LRRC15^pos^fibroblast signature gene expression in rhLAMB3-treated CAFs (n = 3 per group). (**B**) Western blot analysis of LAMB3 level in PANC-1 and MIA PaCa-2 with LRRC15 overexpression. (**C**) Human activated primary CD8^+^ T cells were directly co-cultured with CAFs pretreated with rhLAMB3, and their cytotoxicity was assessed by flow cytometry (n = 3 in each group). (**D, E**) Human CD8^+^ T cells were directly co-cultured with CAFs pretreated with conditioned medium from LAMB3-OE MIA-PaCa-2 (**D**) or PANC-1 (**E**), and their cytotoxicity were assessed by flow cytometry (n = 3 in each group). (**F**) Scatter plot showing the Spearman correlation between *FOSL2* expression in fibroblasts and the relative proportion of Fib_C7_IGF1high among total fibroblasts across samples in the scRNA-seq dataset. Shading represents the 95% confidence interval. (**G, H**) Scatter plots illustrating the Pearson correlation between RNA (**G**) and protein (**H**) level of FOSL2 and features of LRRC15^+^ fibroblasts across individual samples in the TCGA-PAAD and the CPTAC-PDAC cohorts, respectively. (**I**) Western blot analysis of FOSL2 and LRRC15 levels in CAFs with FOSL2 knockdown. siFOSL2-2 was selected for subsequent experiments. (**J**) Dot plot showing scaled average expression of selected genes from the ITGB1/FAK/MAPK/FOSL2 pathway in fibroblasts. Dot size represents the percentage of cells expressing the genes in each group. Color reflects average expression. (**K**) Heatmap displaying the Spearman correlation between *LAMB3* expression in ductal cells and ITGB1/FAK/MAPK/FOSL2 pathway-related gene expression in fibroblasts across samples. (**L**) Scatter plots revealing the Pearson correlation between LRRC15^+^ fibroblasts features and the expression levels of *ITGB1* and *MAPK14* (encoding p38 MAPK) in each sample. (**M, N**) Heatmaps depicting the Pearson correlation between ITGB1/FAK/MAPK/FOSL2 pathway and LAMB3 expression as well as LRRC15^+^ fibroblast features, respectively in the TCGA-PAAD (**M**) and CPTAC-PDAC (**N**) cohorts. (**O**) Western blot analysis of ITGB1 level in CAFs with ITGB1 knockdown, and siITGB1-3 was selected for subsequent assays. Statistical analysis was performed using two-tailed Student’s t test (**A**, **C**, **D**, and **E**). For **A**, **C**, **D**, and **E**, data are shown as mean ± SD. **p* < 0.05, ***p* < 0.01, ****p* < 0.001**.**

**Figure S8. Flow cytometry gating strategy for CD8^+^ T cell analysis in mouse PDAC tumors. Related to Figure 7.**

**Figure S9. Transcriptional characteristics of ductal cells across disease stages. Related to Figure 8.** (**A**) UMAP visualization of ductal cell subsets. (**B**) UMAP plots illustrating the distribution of ductal cell subsets across stages. (**C**) Dot plot showing representative genes for each ductal cell subpopulation. Dot size represents the percentage of cells expressing the genes in each group. Color intensity reflects gene expression levels. (**D**) Heatmap showing GSVA scores for hallmark gene sets in ductal cells across stages. (**E**) Heatmap showing hierarchical clustering of Jaccard similarity between programs across tumoral ductal cells in IPMN tissue samples identified by non-negative matrix factorization. (**F**) Plots depicting the findings after reanalyzing bulk RNA-seq data from Hiraoka *et al.*: Scatter plot (top left) showing the distribution of glycolysis score in epithelium across stages. Box plots (top middle and top right) showing glycolysis score and *SLC2A1* expression level in epithelium. Scatter plots (bottom) showing the Spearman correlation between *LAMB3* expression and glycolytic activity across each sample. Shading represents 95% confidence intervals (UNIN: n = 7; LGD: n = 6; HGD: n = 6; PDAC: n = 3). (**G**) Analogous plots from reanalysis of bulk RNA-seq data from Liffers *et al.*, following the same layout and interpretation as **F** (UNIN: n = 3; LGD: n = 12; HGD: n = 12; cPDAC: n = 4). (**H**) Box plots showing glycolysis score and expression levels of SLC2A1 and LDHA across stages in CPTAC-PDAC cohort (Normal: n = 44; Tumor: n = 105). Statistical analysis was performed using two-tailed Wilcoxon rank-sum test (**F, G,** and **H**).

**Figure S10. Distinct datasets support a strong association between glycolysis and LAMB3 expression.** **Related to Figure 8.** (**A**) Scatter plots showing the Pearson correlation between LAMB3 level and glycolytic activity across samples in the CPTAC-PDAC cohort. (**B**) Heatmap displaying Pearson correlation between glycolytic activity and LRRC15^+^ fibroblast features across stages in the CPTAC-PDAC cohort. (**C**) Scatter plots unveiling the Pearson correlation between *LAMB3* expression and glycolytic activity in the TCGA-PAAD cohort. (**D**) Heatmap displaying the Pearson correlation between glycolytic activity and LRRC15^+^ fibroblasts in the TCGA-PAAD cohort. (**E**) Representative mIHC images showing concurrently low density of SLC2A1^+^LAMB3^+^ epithelial cells and LRRC15^+^ fibroblasts in a patient from the TMA PDAC cohort. DAPI, LRRC15, PDPN, LAMB3, SLC2A1, and PanCK are shown in blue, pink, red, yellow, green, and cyan, respectively. Scale bars: low magnification, 100 μm; high magnification, 25 μm. (**F**) Kaplan-Meier curves showing the overall survival probability based on LAMB3^+^ epithelial cell fraction (left), SLC2A1^+^ epithelial cell fraction (middle), and SLC2A1^+^LAMB3^+^ epithelial cell fraction (right) in the TMA PDAC cohort. (**G, H**) Western blot analysis of HIF-1α levels in MIA PaCa-2 (**G**) and PANC-1 (**H**) with HIF-1α knockdown. siHIF-1α-2 was selected for subsequent assays. Statistical analysis was performed using log-rank test (**F**) and Tarone-Ware test (**F**). **p* < 0.05, ***p* < 0.01, ****p* < 0.001.

**Table S1. Clinical information for the scRNA-seq and spatial transcriptomics cohort**

| Patient ID | Sample ID | Gender | Age | Disease | Surgery |
| --- | --- | --- | --- | --- | --- |
| CN2P03 | CN2P03N | Male | 50 | None | None |
| P02 | P02N, P02I | Male | 49 | PDAC, IPMN | Yes |
| P03 | P03I, P03T | Male | 68 | PDAC, IPMN | Yes |
| P04 | P04I, P04T (P04) | Male | 55 | PDAC, IPMN | Yes |
| P05 | P05I, P05T (P05) | Male | 66 | PDAC, IPMN | Yes |
| P06 | P06I | Male | 71 | PDAC, IPMN | Yes |
| P12 | P12I, P12T | Male | 70 | PDAC, IPMN | Yes |
| P13 | P13I | Female | 83 | IPMN | Yes |
| P15 | P15N, P15T | Male | 45 | PDAC | Yes |
| P16 | P16I, P16T | Male | 57 | PDAC, IPMN | Yes |
| UMP17 | UMP17N | Unknown | Unknown | Unknown | Unknown |
| UMP18 | UMP18N | Unknown | Unknown | Unknown | Unknown |
| UMP19 | UMP19N | Unknown | Unknown | Unknown | Unknown |

**Table S2. Baseline characteristics and clinical outcomes of patients with PDAC treated with combined anti-CD73 and anti-PD-1 immunotherapy at PUMCH**

| Patient ID | Age | Gender | Disease | Prior treatment | Dose level | Best response | Follow-up duration (months) |
| --- | --- | --- | --- | --- | --- | --- | --- |
| #01 | 56 | Male | PDAC | Neoadjuvant chemotherapy + surgery + adjuvant chemotherapy | Reduced-dose anti-CD73 antibody + full-dose anti-PD-1 antibody | iPR | 20 |
| #02 | 60 | Male | PDAC | Neoadjuvant chemotherapy + surgery | Full-dose anti-CD73 antibody + full-dose anti-PD-1 antibody | iSD | 5 |
| #03 | 36 | Female | PDAC | Surgery + adjuvant chemotherapy | Full-dose anti-CD73 antibody + full-dose anti-PD-1 antibody | iPR | 16 |
| #04 | 59 | Male | PDAC | Surgery + adjuvant chemotherapy | Full-dose anti-CD73 antibody + full-dose anti-PD-1 antibody | iPD | 3 |
| #05 | 69 | Female | PDAC | Neoadjuvant chemotherapy + surgery + adjuvant chemotherapy | Full-dose anti-CD73 antibody + full-dose anti-PD-1 antibody | iSD | 7 |
| #06 | 66 | Male | PDAC | Surgery + adjuvant chemotherapy | Full-dose anti-CD73 antibody + full-dose anti-PD-1 antibody | iPD | 2 |
| #07 | 67 | Female | PDAC | Surgery + adjuvant chemotherapy | Full-dose anti-CD73 antibody + full-dose anti-PD-1 antibody | iPD | 5 |

**Table S3. Canonical marker genes of major cell type**

| Major cell types | Canonical marker genes |
| --- | --- |
| Ductal cell | KRT19, CFTR |
| Acinar cell | PRSS1, CLPS, PNLIP, CTRB1 |
| Endocrine cell | CHGA, CHGB, TTR, PPY |
| Tuft cell | AZGP1, AVIL, SH2D6 |
| Fibroblast | PDPN, COL1A1, PDGFRA, LUM, DCN |
| Pericyte_like | RGS5, ACTA2, MYL9 |
| Schwann cell | CDH19, NRXN1, NGFR |
| Endothelial cell | PLVAP, PECAM1, VWF, CD34 |
| T cell | CD3D, CD3E, CD3G, CD2 |
| ILC3&NK cell | NKG7, KLRD1, FCGR3A, NCAM1 |
| B cell | CD79A, BANK1, CD79B |
| Plasma cell | JCHAIN, IGLC2, MZB1 |
| Monocyte | CD14, VCAN, FCN1 |
| Macrophage | CD68, CD80, CD86, C1QA |
| Neutrophil | G0S2, CSF3R |
| Mast cell | TPSB2, KIT, MS4A2 |
| Dendritic cell | CD1C, CLEC10A, FCER1A |

**Table S4. Signature score list**

| Signature names | Gene list |
| --- | --- |
| Fibroblasts |  |
| Normal fibroblast score | APOD, C7, PTGDS, FMO2, PTN, ADH1B, CFD, DPT, CXCL12, SRPX |
| Inflammatory fibroblast score | CXCL12, IL6, IL11, LIF, CCL2, HAS1, HAS2 |
| Myofibroblast score | TAGLN, ACTA2, BGN, MMP11, THY1, MCAM, COL18A1, ADIRF, MYH11, NDUFA4L2, NOTCH3, CSRP2, TINAGL1, RGS5, COL4A1, HIF1A, POSTN, MYL9, HOPX, POSTN, TPM1, TPM2, TGFB1, SMAD2, SNAI1, SOX4, TWIST1, CTHRC1, INHBA, THBS2, FAP |
| LRRC15^pos^myofibroblast score | MMP11, COL11A1, C1QTNF3, CTHRC1, COL12A1, COL10A1, COL5A2, THBS2, AEBP1, LRRC15, ITGA11 |
| Antigen-presenting fibroblast score | CD74, SLPI, SAA2, SAA1, SAA3, CRYAB, GPM6B, CDH19, HLA-DRB1, GAP43, NOV, PLP1, S100B, NRXN1, ITGB8, HLA-DQA1, HLA-DQB1, HLA-DQA2, HLA-DQB2, HLA-DRA, HLA-DPA1, VIM, NFE2L2, PDGFRB, NKAIN4, IRF5, CLU, EZR, ARHGDIB, PTGIS, MSLN, F11R, SLC9A3R1 |
| T cells |  |
| Naive score | CCR7, TCF7, LEF1, SELL, MAL |
| Cytotoxic score | PRF1, IFNG, GNLY, NKG7, GZMB, GZMA, GZMH, KLRK1, KLRB1, KLRD1, CTSW, CST7 |
| Exhaustion score | HAVCR2, CXCL13, CCL3, SIRPG, IFNG, TIGIT, GZMB, PDCD1, PARK7, TNFRSF9, ACP5, CTLA4, RBPJ, MIR155, CXCR6, CD27, FKBP1A, BST2, TPI1, MIR155HG, PTTG1, CD63, SAMSN1, RGS1, CD27-AS1, ITGAE, MIR4632, HLA-DRA, IGFLR1, KRT86, ENTPD1, DUSP4, SIT1, TOX, PHLDA1, CCND2, GPR25, LAYN, PRDX5, SARDH, FASLG, MIR3917, ANXA5, CTSD, PDIA6, RANBP1, FKBP1A-SDCBP2, COTL1, TNFRSF1B, IDH2, CD38, CD82, LAG3, MIR497HG, APOBEC3C, ITM2A, COX5A, IFI35, NDFIP2, TNFRSF18, KRT81, DNPH1, RGS2, HMGN1, DYNLL1, SNRPB, STRA13, SYNGR2, RAB27A, PSMC3, GALM, FABP5, UBE2L6, MYO7A, PRDX3, DDIT4, STMN1, CDK2AP2, VCAM1, SNAP47, PSMB3, ISG15, HLA-DRB5, CKS2, TNIP3, CD7, PSMD4, ATP6V1C2, PSMD8, HLA-DRB6 |
| Exhaustion TF score | SOX4, FOXP3, TOX, TOX2, RBPJ, ZBED2, PRDM1, IKZF4, BATF, STAT3, IFI16 |
| Macrophages |  |
| Angiogenesis score | CCND2, CCNE1, CD44, CXCR4, E2F3, EDN1, EZH2, FGF18, FGFR1, FYN, HEY1, ITGAV, JAG1, JAG2, MMP9, NOTCH1, PDGFA, PTK2, SPP1, STC1, TNFAIP6, TYMP, VAV2, VCAN, VEGFA |
| LYVE1^high^ tissue resident macrophage score | LYVE1, SIGLEC1, FOLR2, MRC1, MSR1, CD209, PDGFB, PDGFC, CCL8, CCL2, F13A1 |
| Extracellular matrix remodeling score | COL1A2, COL3A1, COL6A1, COL5A1, COL4A4, COL10A1, COL17A1, COL18A1, TNC, ELN, SPARC, HAS2, HAS3, LOX, LOXL1, ADAMTS12, MMP2, YAP1 |
| M1 score | NOS2, IL12A, IL12B, IL6, TNF, CD86, IL1B, CXCL9, CXCL10, CXCL11, CXCL12, STAT1, AIM2, IL23 |
| M2 score | MRC1, ARG1, CCL22, CCL17, CCL24, CCL18, CXCR4, NRP1, VEGFA, VEGFB, PDGFB, LLGL1, MMP12, CD209, IL10, WNT5A, ADORA3, STAT6, SOCS3, IRF4, LTA, TFRC, IL4 |
| Tumor ductal cells |  |
| Glycolysis | SLC2A1, HK2, GPI, GAPDH, PGK1, PKM, LDHA |

**Table S5. mIHC panel**

| Antibodies | Source | Catalog number | Dilution | Fluorophore |
| --- | --- | --- | --- | --- |
| LAMB3 | Santa cruz | sc-133178 | 1:50 | opal 520 |
| LRRC15 | Abcam | ab150376 | 1:400 | opal 570 |
| PDPN | Abcam | ab236529 | 1:4000 | opal 620 |
| SLC2A1 | Abcam | ab115730 | 1:2500 | opal 690 |
| PanCK | Dako | IR05361-2CN | Ready to use | opal 780 |
| LRRC15 for mouse | CST | 50546S | 1:200 | opal 520 |

**Table S6. Interference sequences related to siRNA transfection**

| Gene | Knockdown genes with siRNA | Target sequences |
| --- | --- | --- |
| ITGB1 | si-ITGB1-1 | AUCAGAUUCAUCAGAAUGG |
|  | si-ITGB1-2 | GGAACCCUUGCACAAGUGA |
|  | si-ITGB1-3 | GGAUAUUACUCAGAUCCAA |
|  | si-ITGB1-4 | GCGAGUGUGAUAAUUUCAA |
| FOSL2 | si-FOSL2-1 | GGCCCAGTGTGCAAGATTA |
|  | si-FOSL2-2 | GCGCTGTAGTGGTGAAACA |
|  | si-FOSL2-3 | CTGGCGTGATCAAGACCAT |
| HIF-1α | si-HIF-1α-1 | GGAACATGATGGTTCACTT |
|  | si-HIF-1α-2 | CTACCCACATACATAAAGA |
|  | si-HIF-1α-3 | CCAGCAACTTGAGGAAGTA |

**Table S7. shRNA interference sequences used for transfection**

| **shRNA** | **Sequence** |
| --- | --- |
| shLAMB3-1 | GCATGTTGATTGAGCGCTCTTCTCGAGAAGAGCGCTCAATCAACATGCTTTTT |
| shLAMB3-2 | CCGAAGCAAGAAGGAGCAATTCTCGAGAATTGCTCCTTCTTGCTTCGGTTTTTT |
| shLAMB3-3 | GCTGCGCAAGATGAAAGAGATTTCAAGAGAATCTCTTTCATCTTGCGCAGCTTTTTT |
| shCtrl | TTCTCCGAACGTGTCACGT |

**Table S8. Antibodies for Western blot**

| Antibodies | Source | Catalog number | dilution | Species | KDa |
| --- | --- | --- | --- | --- | --- |
| LRRC15 | Abcam, UK | ab150376 | 1:1000 | Rabbit | 64 |
| GAPDH | Abcam, UK | ab8245 | 1:1000 | Mouse | 35 |
| LAMB3 | Santa cruz | sc-133178 | 1:1000 | Mouse | 120 |
| LAMB3 | Abcam, UK | ab150385 | 1:1000 | Rabbit | 120 |
| β-actin | proteintech | 66009-1-Ig | 1:20000 | Mouse | 42 |
| HIF-1α | Abcam, UK | ab179483 | 1:1000 | Rabbit | 110 |
| FOSL2 | Cell Signaling Technology, USA | #19967S | 1:1000 | Rabbit | 35-45 |
| FAK | Cell Signaling Technology, USA | #71433 | 1:1000 | Rabbit | 125 |
| pFAK(Tyr397) | Abcam, UK | ab39967 | 1:1000 | Rabbit | 119 |
| Src | Cell Signaling Technology, USA | #2109 | 1:1000 | Rabbit | 60 |
| pSrc(Tyr416) | Cell Signaling Technology, USA | #6943 | 1:1000 | Rabbit | 60 |
| p38 MAPK | Cell Signaling Technology, USA | #8690 | 1:1000 | Rabbit | 43 |
| p-p38 MAPK (Thr180/Tyr182) | Cell Signaling Technology, USA | #4511 | 1:1000 | Rabbit | 43 |
| c-jun | Abcam, UK | ab40766 | 1:1000 | Rabbit | 39 |
| ITGB1 | Abcam, UK | ab52971 | 1:1000 | Rabbit | 140 |
| goat anti-rabbit IgG | ZSGB-BIO, China | ZB-2301 | 1:5000 | Goat | - |
| goat anti-mouse IgG | ZSGB-BIO, China | ZB-2305 | 1:5000 | Goat | - |

**Table S9. Relevant primer sequences for qRT-PCR**

| Gene | Primer sequences | |
| --- | --- | --- |
| LRRC15 | Forward | TGCCCTAGCGAGTGTACCT |
|  | Reverse | GATGTGCGTGTTGAGGATCTG |
| GAPDH | Forward | GCAAATTCCATGGCACCGTCAA |
|  | Reverse | CATGGTGGTGAAGACGCCAGT |
| C1QTNF3 | Forward | GAGAAGGGCGACAAAGGTGA |
|  | Reverse | GATTGCTGAAGTGGGTTGCC |
| CTHRC1 | Forward | TTTCCAGGGGCTCATCTGTG |
|  | Reverse | TTGAATCCATCCCGACCTGG |
| COL10A1 | Forward | AACTCCCAGCACGCAGAATC |
|  | Reverse | CTTGGTGTTGGGTAGTGGGC |
| MMP11 | Forward | TCTTCCCCAAGACTCACCGA |
|  | Reverse | GTGGCCAAATTCATGGGCTG |
| COL5A2 | Forward | AGAGCCCACAGCTGACTTC |
|  | Reverse | TGAGGAGAGGTCTTGCTTCC |
| AEBP1 | Forward | GAGAGGATTGAGCCTCCTGTG |
|  | Reverse | CCTTCTCTTCGTCTGTCTGGC |
| COL11A1 | Forward | CAATAGCACAGACGGAGGCAA |
|  | Reverse | GGCTCATTTGTCCCAGAAACA |
| THBS2 | Forward | CCCTTGGATCCCAAAGGGAC |
|  | Reverse | CCACTGAAGTCCACAGACCC |
| COL12A1 | Forward | CACAAGCTCCCAAAAGTGGC |
|  | Reverse | TCCCCTGTGGAAGGCTGATA |
| ITGA11 | Forward | CCTCCGAAGCCCTCCTAAAAT |
|  | Reverse | AGAGCAAGCAATGACCCAGC |

**Table S10. Relevant primer sequences for ChIP-qPCR**

| Gene | Primer sequences | |
| --- | --- | --- |
| LRRC15-ChIP-Primer#1 | Forward | GGATGGAAGAGGAAAAAGAGGG |
|  | Reverse | CTCCTGGTCTCATGATTCTCG |
| LRRC15-ChIP-Primer#2 | Forward | TGGGCTCCTGTTATTTAGTGG |
|  | Reverse | TAGTTGCTTGGGTGGCTTGTC |
| GAPDH | Forward | GCAAATTCCATGGCACCGTCAA |
|  | Reverse | CATGGTGGTGAAGACGCCAGT |
